# HIV Protease Inhibitors Disrupt Lipid Metabolism by Activating Endoplasmic Reticulum Stress and Inhibiting Autophagy Activity in Adipocytes

**DOI:** 10.1371/journal.pone.0059514

**Published:** 2013-03-22

**Authors:** Beth S. Zha, Xiaoshan Wan, Xiaoxuan Zhang, Weibin Zha, Jun Zhou, Martin Wabitsch, Guangji Wang, Vijay Lyall, Phillip B. Hylemon, Huiping Zhou

**Affiliations:** 1 Department of Microbiology and Immunology, School of Medicine, Virginia Commonwealth University, Richmond, Virginia, United States of America; 2 School of Pharmacy, Wenzhou Medical College, Wenzhou, Zhejiang, P.R. China; 3 Key Laboratory of Drug Metabolism and Pharmacokinetics, China Pharmaceutical University, Nanjing, Jiangsu, P.R. China; 4 Division of Pediatric Endocrinology and Diabetes, University of Ulm, Ulm, Germany; 5 Department of Physiology and Biophysics, School of Medicine, Virginia Commonwealth University, Richmond, Virginia, United States of America; 6 McGuire Veterans Affairs Medical Center, Richmond, Virginia, United States of America; The University of New South Wales, Australia

## Abstract

**Background:**

HIV protease inhibitors (PI) are core components of Highly Active Antiretroviral Therapy (HAART), the most effective treatment for HIV infection currently available. However, HIV PIs have now been linked to lipodystrophy and dyslipidemia, which are major risk factors for cardiovascular disease and metabolic syndrome. Our previous studies have shown that HIV PIs activate endoplasmic reticulum (ER) stress and disrupt lipid metabolism in hepatocytes and macrophages. Yet, little is known on how HIV PIs disrupt lipid metabolism in adipocytes, a major cell type involved in the pathogenesis of metabolic syndrome.

**Methodology and Principal Findings:**

Cultured and primary mouse adipocytes and human adipocytes were used to examine the effect of frequently used HIV PIs in the clinic, lopinavir/ritonavir, on adipocyte differentiation and further identify the underlying molecular mechanism of HIV PI-induced dysregulation of lipid metabolism in adipocytes. The results indicated that lopinavir alone or in combination with ritonavir, significantly activated the ER stress response, inhibited cell differentiation, and induced cell apoptosis in adipocytes. In addition, HIV PI-induced ER stress was closely linked to inhibition of autophagy activity. We also identified through the use of primary adipocytes of CHOP^−/−^ mice that CHOP, the major transcriptional factor of the ER stress signaling pathway, is involved in lopinavir/ritonavir-induced inhibition of cell differentiation in adipocytes. In addition, lopinavir/ritonavir-induced ER stress appears to be associated with inhibition of autophagy activity in adipocytes.

**Conclusion and Significance:**

Activation of ER stress and impairment of autophagy activity are involved in HIV PI-induced dysregulation of lipid metabolism in adipocytes. The key components of ER stress and autophagy signaling pathways are potential therapeutic targets for HIV PI-induced metabolic side effects in HIV patients.

## Introduction

The development of HIV protease inhibitors (PIs) was one of the most significant advances of the past two decades for controlling HIV infection. Formulation of Highly Active Antiretroviral Therapy (HAART) with the inclusion of HIV PIs in patient treatment has had a profound impact on the clinical history of HIV. However, HAART has been linked to cardiovascular complications and metabolic syndrome in HIV-1 patients. It has been well-documented that HIV PIs specifically induce many of these deleterious effects including early induction of insulin resistance, dysregulation of lipid metabolism, and inflammation, all of which are cornerstones of cardiovascular and metabolic diseases [1], [2].

During the last decade, an extensive effort has been put forth to study the mechanism underlying HIV PI-induced side effects. Both *in vitro* and *in vivo* animal studies from our laboratory and others’ have linked the activation of endoplasmic reticulum (ER) stress to HIV PI-induced cell apoptosis, dyslipidemia, inflammation, and insulin resistance in several metabolically important cell types including hepatocytes, macrophages, and adipocytes [3]–[7]. The contribution of adipocytes to the pathogenesis of cardiovascular and metabolic diseases is becoming widely appreciated. Adipocytes are not only storage units for triglycerides, but also influence systemic lipid homeostasis through the production and release of adipocyte-specific and adipocyte-enriched hormonal factors, inflammatory mediators and adipokines. Disruption of cellular homeostasis of adipocytes can be central in the inflammatory state, insulin resistance, dyslipidemia, and altered body morphology [8]–[14]. HIV PIs have surprisingly similar effects in HIV-infected patients [15]–[19]. Several studies have reported that HIV PIs inhibit adipocyte differentiation, alter the expression of adipocytokines, and induce insulin resistance [20]–[24].

Autophagy is an intracellular protein degradation system required for normal turnover of cellular components and for the starvation response and plays an important physiological role in eukaryotic cells [25]. It has been recently discovered that autophagy activation is closely linked to ER stress and the unfolded protein response (UPR) pathways [26]. Autophagy is not only a critical regulator of hepatic lipid metabolism, but also plays an important role in regulation of adipose lipid storage and adipocyte differentiation [25], [27], [28]. However, little is known about how ER stress and autophagy interact in HIV PI-induced dysregulation of lipid metabolism in adipocytes.

In this study, we examined the effect of current clinically relevant HIV PIs on ER stress and autophagy activation both in cultured mouse and human adipocytes and primary mouse adipocytes, and further identified the potential link between these two important cellular pathways in HIV PI-induced dysfunction of adipocytes.

## Materials and Methods

*Materials:* Antibodies against C/EBP homologous protein (CHOP), activating transcription factor-4 (ATF-4), X-box-binding protein-1 (XBP-1), lamin B, ATG5, ATG7 and horseradish peroxidase (HRP)-conjugated donkey anti-goat IgG were from Santa Cruz Biotechnology (Santa Cruz, CA). LC3B antibody was obtained from Cell Signaling (Danvers, MA). Bio-Rad protein assay reagent, Criterion XT Precast Gel, HRP-conjugated goat anti-rabbit and anti-mouse IgG, p62 and Precision Plus Protein Kaleidoscope Standards were obtained from Bio-Rad (Hercules, CA). HIV protease inhibitors, amprenavir (APV), indinavir (IDV), atazanavir (AZV), ritonavir (RTV), lopinavir (LPV), nelfinavir (NEV), saquinavir (SQV), darunavir (DAV), and tipranavir (TRV), were obtained through the AIDS Research and Reference Reagent Program, Division of AIDS, NIAID, NIH. Thapsigargin (TG), dimethyl sulfoxide molecular biology grade (DMSO), 3-Isobutyl-1-methylxanthine (IBMX), rosiglitazone, phosphatase inhibitor mix, dexamethasone, hydrocortisone, Nile red, and Oil Red O were obtained from Sigma Aldrich (St. Louis, MO). Chemiluminescence Reagent was from PerkinElmer Life Sciences. RNeasy MinElute Cleanup Kit was from Qiagen.

### Culture of Adipocytes

3T3-L1 murine pre-adipocytes were obtained from ATCC (Manassas, VA). Cells were maintained in DMEM with 10% newborn calf serum and 1% penicillin-streptomycin (P-S) at 37°C with 5% CO_2_ until confluence. Differentiation was induced by replacing media with DMEM 10% fetal bovine serum (FBS), 1% P-S, 0.5 mM IBMX, 0.8 µM insulin, and 1 µM dexamethasone. After three days, the induction medium was removed and replaced by DMEM 10% FBS, 1% P-S, and 0.8 µM insulin for two days, followed by DMEM 10% FBS/1% P-S for 3–4 more days. Mature adipocytes were used in experiments after 80% of cells visually appeared differentiated (minimum of 8 total days).

Human Simpson-Golbai-Behmel Syndrome (SGBS) pre-adipocytes were a kind gift from Dr. Martin Wabitsch (University of Ulm, Germany) [29]. SGBS cells were maintained in DMEM/F12 with 10% FCS/1% P-S, 33 mM biotin, and 17 mM pantothenate. Cells were induced to differentiate at confluence in serum free DMEM/F12 with 0.01 mg/mL transferrin, 2×10^?−^8 M insulin, 1×10^−7^ M cortisol, and 0.2 nM T3. For the first 3 days, this media was also supplemented with 25 nM dexamethasone, 500 µM IBMX, and 2 µM rosiglitazone, then these three components were not used for the rest of differentiation. Cells were used in experiments after 80% of cells visually appeared differentiated (average 2 weeks).

### Western Blot Analysis

Total cell lysate (LC3 and p62) or nuclear proteins (UPR activation) were prepared and used for Western blot analysis as previously described [3], [30]. Membranes were incubated with primary antibodies in 2.5% milk-TBS for CHOP, XBP-1, ATF-4, lamin B, p62 or β-Actin, and in 5% BSA-TBST for LC3. Immunoreactive bands were detected using horseradish peroxidase-conjugated secondary antibody and chemiluminescence. The density of the immunoblot was analyzed using either Image J or Quantity One (Biorad).

### RNA Isolation and Quantitative Real-time RT-PCR

Total cellular RNA was isolated from 3T3-L1 pre-adipocytes and mature adipocytes after treatment using QIAGEN RNeasy MinElute Kit. Total RNA (2 μg) was used for first-strand cDNA synthesis using a High-Capacity cDNA Reverse Transcription Kit (Applied Biosytems). The mRNA levels of CHOP and ATF-4 were quantified using the following primers: CHOP forward primer: 5′GTCCCTGCCTTTCACCTTGG3′; CHOP reverse primer: 5′GGTTTTTGATTCTTCCTCTTCG 3′; ATF-4 forward primer: 5′C CTAGGTCTCTTA GATGACTATCTGGAAG3′, ATF-4 reverse primer: 5′CCAGGTCATCCATTCGAAAC AGAGCATCG3′; β-actin forward primer: 5′ ACCACACCTTCTACAATGAG 3′; β-actin reverse primer: 5′ ACGACCA GAGGCATACAG 3′. iQ™ SYBR Green Supermix (Bio-Rad) was used as a fluorescent dye to detect the presence of double-stranded DNA. The mRNA levels of target genes were normalized using β-actin mRNA as an internal control. The ratio of normalized mean value for each treatment group to vehicle control (DMSO) was calculated.

### Analysis of Apoptosis by Annexin V and Propidium Iodine Staining

Cells were treated with individual HIV PIs for 24 h and stained with Annexin V-FITC and propidium iodide using BD ApoAlert Annexin V kit according to the protocol recommended by the manufacturer. Stained cells were further analyzed by two-color flow cytometry. Annexin V-FITC and propidium iodide emissions were detected in the FL1 and FL3 channels respectively of a Cytomics FC 500 flow cytometer (Beckman Coulter, Fullerton, CA). At least 20,000 cells were analyzed in each treatment group.

### Assay of ER Calcium Pools

Non-differentiated 3T3-L1 cells were grown on 22×30-mm coverslips and treated with individual HIV PIs for 24 h. ER calcium stores were analyzed using Fura-2 AM as previously described [3]. Fluorescence images (510-nm emission after alternate 340- and 380-nm excitation) were collected at 15-ms intervals before and after addition of TG. The 340∶380 ratios of 20 individual cells in these images were analyzed using TILLvisION version 3.1 imaging software [3].

### Nile Red and Oil Red O Staining of Intracellular Lipid

3T3-L1 cells were plated on 22×22-mm glass coverslips in 6-well plates until confluence. Cells were treated with HIV PIs while concurrently being induced to differentiate. After 8 days, cells were fixed with 3.7% formaldehyde in PBS for 30 min followed by two washes with PBS. The cells were stained with 0.2% Oil Red O in 60% 2-propanol for 15 min or Nile red (100 ng/ml) for 10 min and washed three times with PBS as previously described [31], [32]. The images of Oil Red O staining were taken with a microscope (Olympus, Tokyo, Japan) equipped with an image recorder under a 40 × lens. Images of Nile red staining were obtained under a 40 × objective using an FITC filter on a fluorescent microscope (Olympus, Center Valley, PA).

### Quantitative Analysis of Lipid Droplets

3T3-L1 cells were plated on 22×22-mm glass coverslips in 6 well plates until confluence. Cells were treated with HIV PIs while concurrently being induced to differentiate. After 14 days, cells were fixed with 3.7% formaldehyde in PBS for 30 min followed by two washes with PBS. The coverslips were mounted on glass slides and images of cells were obtained using a 40 × objective of an upright light microscope Motic BA200. Images were processed using a previously published custom-made MATLAB (MathWorks) code [33]. The lipid droplet number, areas, and % area occupied by lipid droplet were then determined.

### Isolation of Primary Mouse Adipocytes

Both wild type and CHOP^−/−^ mice with C57BL/6J background (8–10 weeks old) were used to obtain primary adipose cells. After euthanization, the gonadal fat pad was excised from mice and placed in 37°C Krebs-Ringer-Hepes (KRH) buffer (1 mM CaCl_2_, 1.2 mM MgSO_4_, 1.2 mM KH_2_PO_4_, 130 mM NaCl, 1.4 mM KCl, 2 mM Pyruvic Acid, 20 mM HEPES with 4 mM NaHCO_3_,) with 0.83% BSA. Tissue was minced until all fragments were no bigger than 1 mm and placed in 1 mg/mL Type I collagenase (Worthington Biochemical) in KRH, and digested for 30 minutes in a rotating water incubator at 37°C. Suspension was filtered, and washed in fresh KRH to remove bound collagenase. Solution was centrifuged at 100×g for 5 minutes and cell pellet was resuspended in DMEM containing 10% FBS, 1% P-S, 33 µM Biotin, 100 µM ascorbic acid, 4 nM insulin and 8.3 mM L-glutamine and plated on 6-well plates. After confluence, cells were induced to differentiate by adding 1 μg/mL insulin, 1 μM dexamethasone, 0.5 mM IBMX, and 1 µM rosiglitazone to media for 48 h. After two days, media was supplemented with insulin and rosiglitazone alone. Cells were thereafter cultured with base media for 6–8 more days for full differentiation to occur.

### Transmission Electron Microscopy (TEM)

3T3-L1 cells were plated on Permanox Quantity dishes (Nalgene Nunc International, Rochester NY), and treated with HIV PIs for 24 and 48 h. Cells were rinsed with PBS and fixed with 2% glutaraldehyde for 1 hour, rinsed in 0.1M cacodylate buffer, and fixed for another hour with 1% osmium tetroxide in 0.1M cacodylate buffer. Samples were further washed, dehydrated in gradient ethanol and infiltrated with a 50/50 mixture of 100% ethanol/PolyBed 812 resin for overnight, and further infiltrated with pure PolyBed. Samples were embedded using fresh PolyBed 812 and polymerized in a 60°C oven for two days. Samples were sectioned with a Leica EM UC6i Ultramicrotome (Leica Microsystems) and stained with 5% uranyl acetate and Reynold’s Lead Citrate, followed by scoping using a JEOL JEM-1230 TEM (JEOL USA) with a Gatan Ultrascan 4000 digital camera (Gatan Inc, Pleasanton CA). Morphometry was based on previously published literature [34]. Electron microscope primary images were obtained at 1000 to 12,000 ×. For each treatment, magnification 4,000× was obtained 4 times in 15 different cells. The cytoplasmic volume fraction of autophagosomes was obtained by point counting with 1.5 cm spacing, and using the equation of points falling on autophagosomes divided by points falling on cytoplasm.

### Monitoring the Autophagy Formation Using GFP-LC3

GFP-LC3 under SV promoter in the retroviral vector pBABE was purchased from AddGene. Retroviral particles were constructed in 293-FT cells by cotransfection with 1 μg pBABE-puro-GFP-LC3, 0.1 μg pCMV-VSV-G, and 0.9 μg pMDLg/pREE using CaCl_2_ and HEPES mixture. Retroviral particles were harvested from culture supernatants 72 h after transfection and further concentrated with 8.5% PEG 6000 containing 0.4 M NaCl. 3T3-L1 cells were infected with retrovirus in the presence of 8 μg/mL of polybrene for 48 h. Successfully infected cells were selected with puromycin (5 μg/ml).

### Analysis of Long-lived Protein Degradation

3T3L1 cells were plated on 12-well plates for overnight. The cell media was removed and replaced by 1 mL DMEM containing ^14^C-Valine (0.2 Ci/ml) adjusted to 62.4 µM final concentration using unlabeled valine and incubated at 37°C for overnight. After removing the labeling medium, the cells were washed with 1 mL of DMEM containing 62.4 μM valine, and then incubated with DMEM containing 10 mM valine for 2 hours to chase out short-lived protein. Cells were treated with HIV PIs with or without autophagy inhibitor, 3-methyladenine (10 mM) for 24 h. At the end of treatment, the radioactivity of the culture media and total cellular lysate was determined as described previously [35].

### Statistical Analysis

All of the experiments were repeated at least three times and the results were expressed as mean ± SE. One-way ANOVA was used to analyze the differences between sets of data using GraphPad Prism (GraphPad, San Diego, CA). A value of p<0.05 was considered statistically significant.

## Results

### HIV PIs Induce ER Stress and Activate the UPR in Adipocytes

HIV PIs, especially LPV and RTV, have been reported to disrupt lipogenesis, induce insulin resistance, and inhibit differentiation in both mouse and human adipocytes [16],[20],[36]–[39]. These studies suggest that disruption of mitochondria function and activation of oxidative stress contribute to HIV PI-associated adverse effect on adipocytes. Our previous studies indicate that activation of ER stress plays a critical role in HIV PI-induced dysregulation of lipid metabolism in macrophages and hepatocytes [3], [40]. In order to determine whether HIV PIs have the similar effects on the UPR activation in adipocytes as they do in other cell types, mouse 3T3-L1 pre- and mature adipocytes were treated with nine available HIV PIs for various time periods (1–24 h) and the protein levels of UPR-specific genes, CHOP, ATF-4, and XBP-1, were detected by Western Blot analysis. Similar to our previous observations, HIV PIs differentially induced UPR activation in pre-adipocytes and mature adipocytes. LPV, RTV, SQV, NEV, and IDV induced significant activation of the UPR, while APV, DAV and TRV only had modest or no activation in adipocytes (Supplementary Figure 1 and Figure 2 in File S1). The most significant activation of the UPR induced by LPV, RTV, and LPV/RTV was observed between 4 to 6 h (Supplementary Figure 3 in File S1). We therefore split these HIV PIs into two groups: ER stress inducers and non-inducers. Interestingly, those in the non-inducer group have much lower incidences of inducing dyslipidemia in patients compared to those in the inducer group [41], [42].

**Figure 1:**
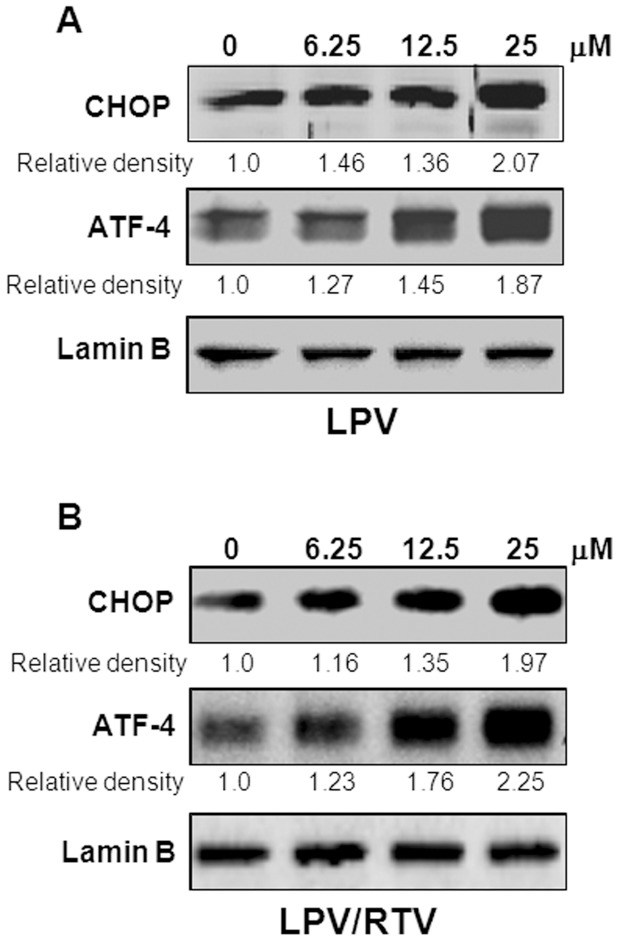
Effect of HIV PIs on the UPR activation in mouse pre-adipocytes. Representative immunoblots against CHOP, ATF-4, and lamin B from the nuclear extracts of mouse 3T3L1 cells treated with different concentrations of HIV PIs for 6 h are shown. **A)**. lopinavir (LPV). **B)**. lopinavir/ritonavir (LPV/RTV = 4∶1). The density of immunoblot was determined by Image J. Relative protein levels of CHOP and ATF-4 were normalized using Lamin B as a loading control.

**Figure 2:**
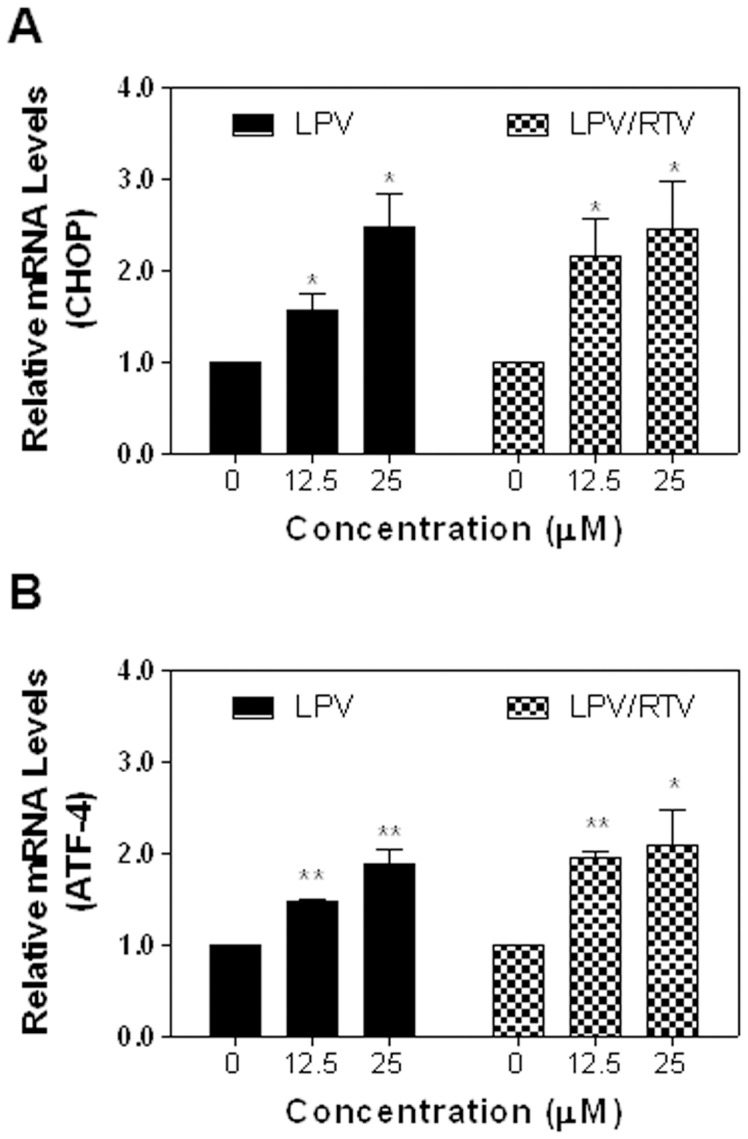
Effect of HIV PIs on UPR activation in differentiated mouse adipocytes. Differentiated 3T3-L1 cells were treated with different concentrations of LPV, or LPV/RTV for 6 h. Total cellular RNA was isolated. The mRNA levels of CHOP and ATF-4 were quantified by real-time RT-PCR and normalized using internal control β-actin. Values are mean ± SE of three independent experiments. Statistical significance relative to vehicle control, *p<0.05, and **p<0.01.

**Figure 3:**
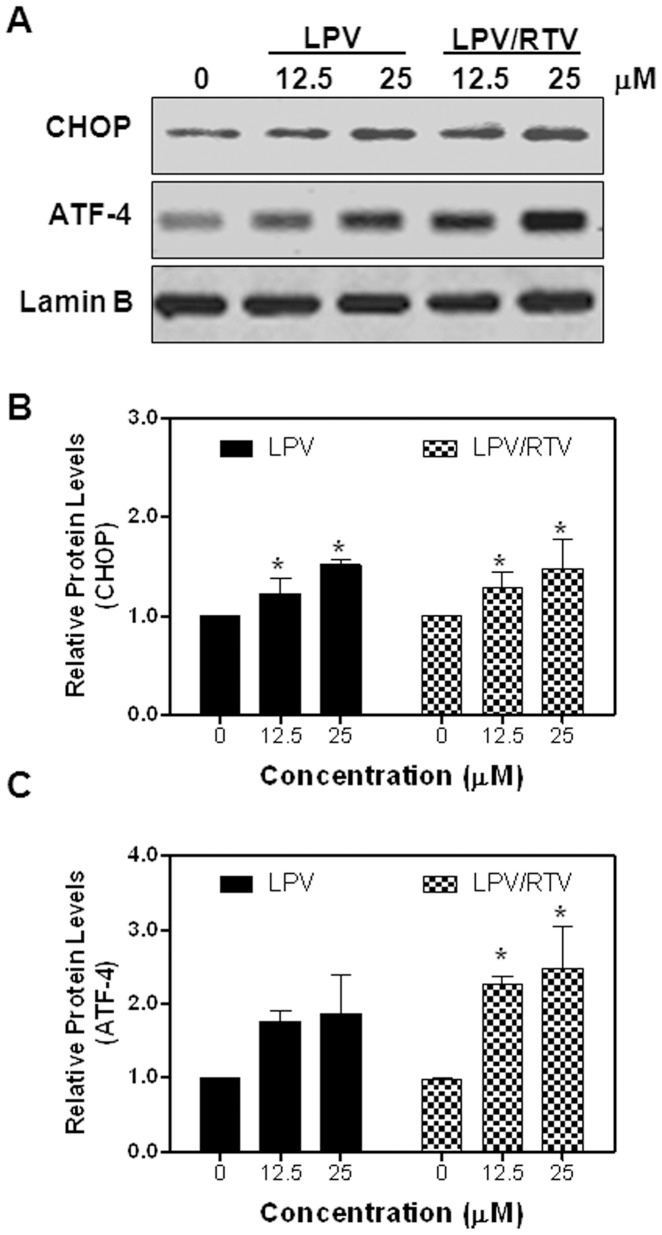
Effect of HIV PIs on UPR activation in differentiated mouse adipocytes. **A)** Representative immunoblots against CHOP, ATF-4 and Lamin B from nuclear extracts of mouse differentiated 3T3L1 cells treated with different concentrations of LPV and LPR/RTV for 6 h are shown. **B–C)** The density of immunoblot was determined by Image J. Relative protein levels of CHOP and ATF-4 were normalized using Lamin B as a loading control. Values are mean ± SE of four independent experiments. Statistical significance relative to vehicle control, *p<0.05.

**Figure 4:**
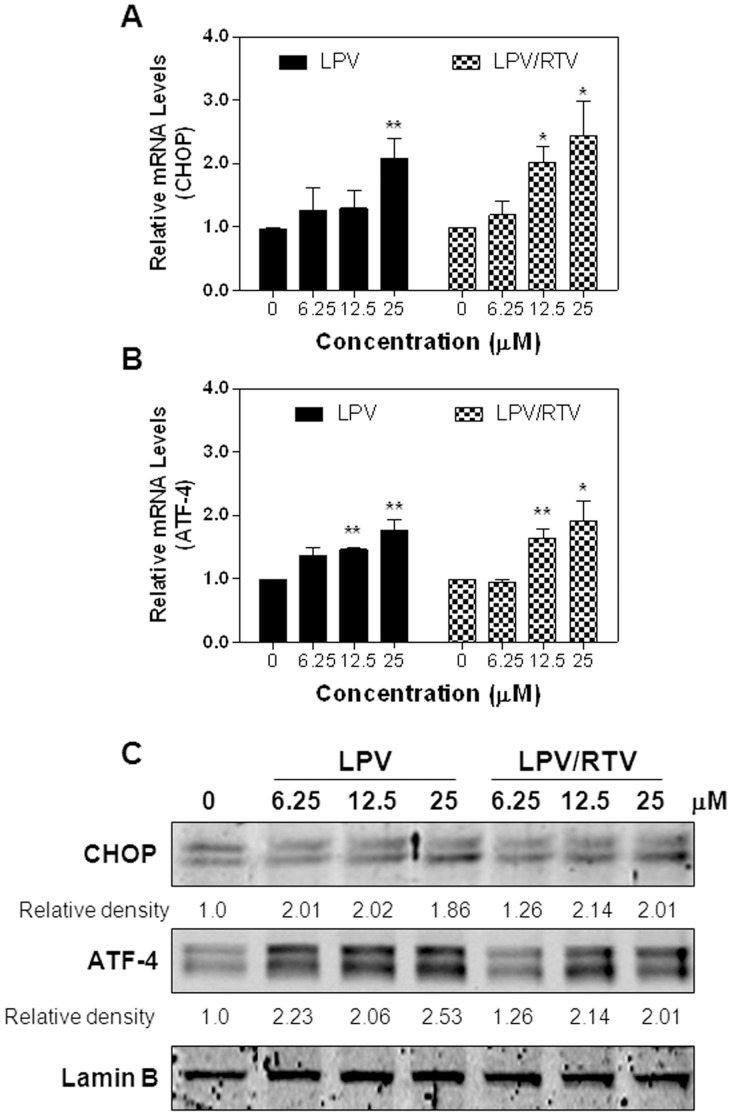
Effect of HIV PIs on the UPR activation in differentiated human adipocytes. **A–B).** Relative mRNA levels of CHOP and ATF-4 from differentiated human SGBS cells treated with different concentrations of LPV or LPV/RTV for 6 h and analyzed by real-time RT-PCR. β-Actin was used as an internal control. Values are mean ± SE of three independent experiments. Statistical significance relative to vehicle control, *p<0.05, **p<0.01. **C).** Representative immunoblots against CHOP, ATF-4 and Lamin B from nuclear extracts of differentiated human SGBS cells treated with different concentrations of LPV or LPV/RTV for 6 h are shown. The density of immunoblot was determined by Image J. Relative protein levels of CHOP and ATF-4 were normalized using Lamin B as a loading control.

Based on the most current guidelines of US Department of Health and Human Services for Use of Antiretroviral Agents in HIV-1-infected Adults and Adolescents, HIV PIs are continuously listed as key components of preferred HAART regimens, and will continue to be important drugs for the foreseeable future. With the ability to maintain viral load suppression superior to some other PIs, LPV co-formulated with RTV (4∶1) has remained a frequently used treatment in the clinic. As LPV/RTV is also in the ER stress inducer group and is known to induce metabolic side effects in the clinic, we further examined the effects of LPV and LPV/RTV on the UPR activation in pre-adipocytes and mature adipocytes. Our studies focused on known physiological concentrations of HIV PIs between 5–25 μM. As shown in Figure 1, LPV and LPV/RTV dose dependently induced CHOP and ATF-4 expression in mouse pre-adipocytes, but had no significant effect on XBP-1 expression (data not shown). Similarly, LPV and LPV/RTV also dose-dependently increased CHOP and ATF-4 expression at both mRNA levels and protein levels in mature mouse adipocytes (Figure 2 and 3). We further confirmed these findings in human SGBS adipocytes. As shown in Figure 4, both LPV and LPV/RTV increased CHOP and ATF-4 expression in differentiated human adipocytes. In addition, RTV alone also significantly activated the UPR in non-differentiated and differentiated mouse adipocytes as well as human adipocytes (Supplementary Figure 4 in File S1).

### HIV PIs Induce Cell Death in Adipocytes

**Figure 5:**
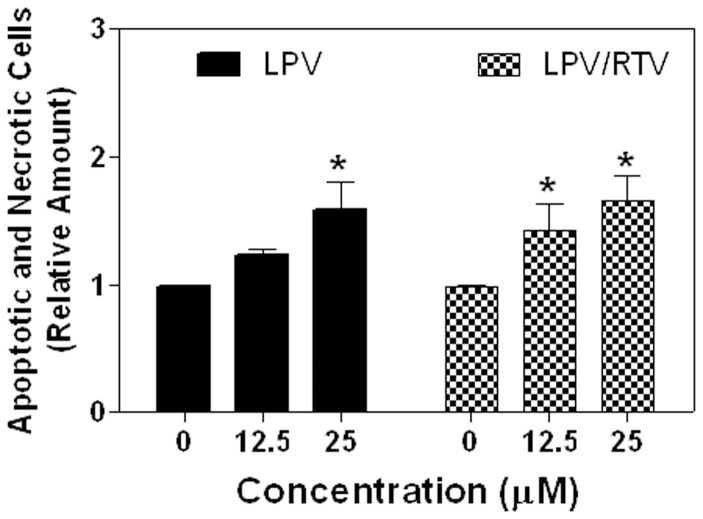
HIV PIs induce cell death in differentiated 3T3-L1s. Differentiated 3T3-L1 cells were treated with different concentrations of HIV PIs or vehicle control (DMSO) for 24 h, and then stained with Annexin V-FITC/propidium iodide. The percentages of apoptotic and necrotic cells were analyzed by flow cytometry. Relative amount of apoptotic and necrotic cells compared to vehicle control (which was set as 1) was calculated. Values are the mean ± SE of four independent experiments. Statistical significance relative to vehicle control, *p<0.05.

We have previously shown that HIV PI-induced ER stress is correlated to the induction of cell apoptosis in macrophages and hepatocytes at clinically relevant concentrations [3], [40]. We further examined whether LPV and LPV/RTV would have a similar affect in adipocytes, which could potentially account for the associated dysregulation of lipid metabolism. Differentiated 3T3-L1s were treated with different concentrations of LPV, LPV/RTV or vehicle control DMSO for 24 h. Cells were collected, stained with annexin V-FITC and propidium iodide, and analyzed by flow cytometry. As shown in Figure 5, LPV and LPV/RTV promoted cell death in differentiated 3T3-L1s.

### Effects of HIV PIs on Intracellular Lipid Accumulation in Adipocytes

**Figure 6:**
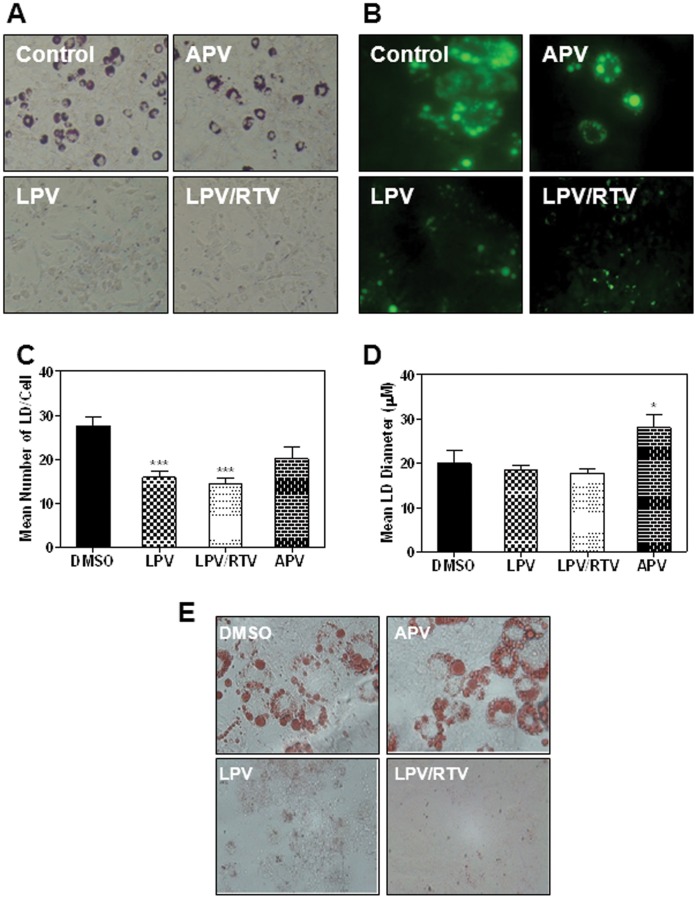
Effect of HIV PIs on adipocyte differentiation. **A–B)** 3T3-L1 cells were induced to differentiate while concurrently treated with different HIV PIs (12.5 µM). After 8 days, cells were fixed and stained with Oil Red O and Nile red. Representative images of Oil Red O staining and Nile red staining are shown. **C–D)** 3T3-L1 cells were induced to differentiate while concurrently treated with different HIV PIs (12.5 µM). After two weeks, cells were fixed and 40 × images were processed using MATLAB. The number and size of lipid droplets were measured. Values are mean ± SE for three independent experiments. Statistical significance relative to vehicle control, ***p<0.001. **E)** Human SGBS cells were induced to differentiate while concurrently treated with different HIV PIs (12.5 µM) for 10 days. The intracellular lipid was stained with Oil Red O. Representative images from three individual experiments are shown.

Previous studies have shown that HIV PIs can affect adipocyte differentiation, but with contradictory results [23], [43], [44]. Our previous studies also showed that individual HIV PIs had different effects on lipid metabolism in macrophages and hepatocytes. APV had little effect, but LPV and RTV had the most significant effect on lipid metabolism [3], [40]. In order to determine the effect of APV, LPV, and LPV/RTV (4∶1) on adipocytes differentiation, murine pre-adipocytes were induced to differentiate while concurrently treated with 12.5 μM HIV PIs for 8 days. The intracellular lipid was stained using Oil Red O and Nile red. As shown in Figure 6A and B, APV had little effect on lipid accumulation, however, LPV and LPV/RTV significantly inhibited lipid accumulation. Similar results were obtained with human SGBS cells stained with Oil Red O (Figure 6E). To increase accuracy and avoid subjectivity, we also quantitated both the number and size of lipid droplets that accumulated in 3T3-L1s when induced to differentiate in the presence of HIV PIs using a MATLAB program as described previously [33]. As shown in Figure 6C and D, LPV and LPV/RTV significantly reduced the number of lipid droplet (LD), but had no significant effect on the size of LD. APV had no effect on the number of LD, but increased the size of LD. These results indicated that ER stress activators, LPV and LPV/RTV, inhibit essential LD formation during adipocyte differentiation.

**Figure 7:**
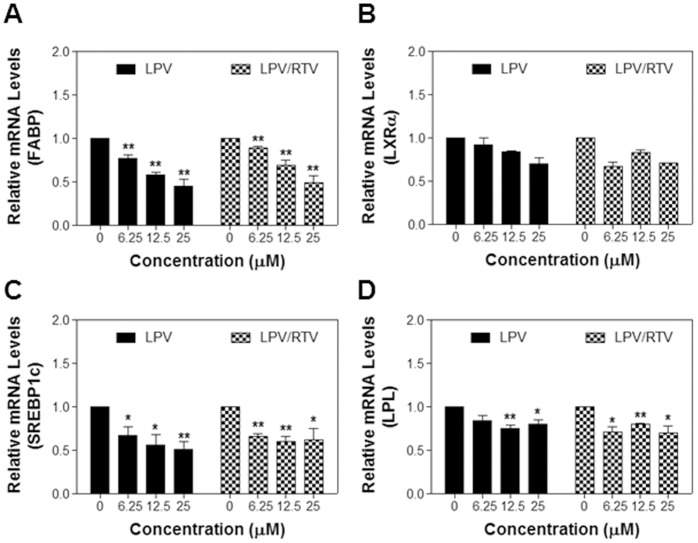
Effect of HIV PIs on the expression of essential genes involved in lipid metabolism in differentiated 3T3-L1 cells. Differentiated 3T3-L1 cells were treated for 6 h with increasing concentrations of LPV or LPV/RTV. Total cellular RNA was isolated and the mRNA levels of LPL, LXRα, FABP, and SREBP-1c were quantified by real-time RT-PCR and normalized using internal control β-Actin. Values are mean ± SE of three independent experiments. Statistical significance relative to vehicle control, *p<0.05, and **p<0.01.

To further determine whether HIV PI-induced inhibition of LD formation is correlated to the inhibition of the key genes involved in adipocyte differentiation, we determined the effect of LPV and LPV/RTV on the mRNA expression of sterol regulatory element-binding protein-1c (SREBP-1c), lipoprotein lipase (LPL), fatty acid binding protein (FABP), peroxisome proliferator-activated receptor gamma (PPARγ), and liver X receptor alpha (LXRα) in differentiated 3T3L1 cells. As shown in Figure 7, LPV and LPV/RTV significantly inhibited FABP, SREBP-1c and LPL mRNA expression, but no effect on LXRα and PPARγ (data not shown).

### Effect of CHOP on HIV PI-induced Alterations of Intracellular Lipid Accumulation in Adipocytes

**Figure 8:**
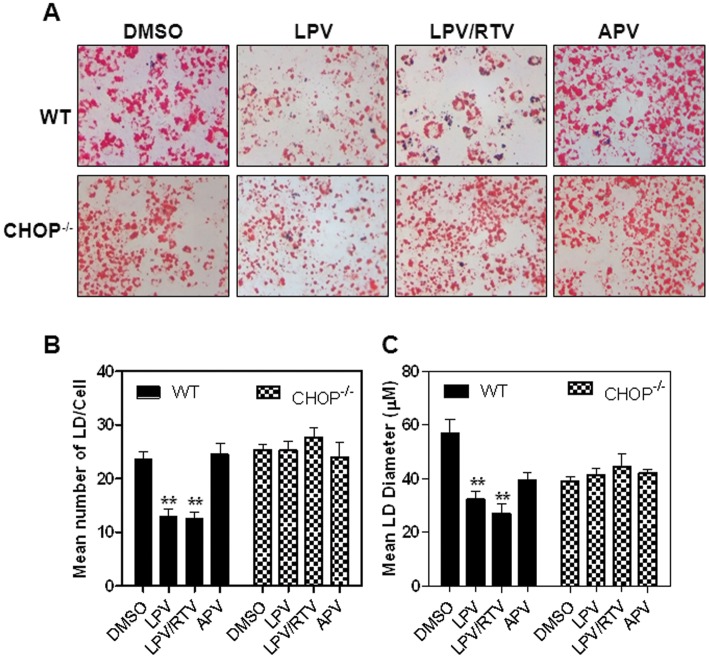
Effect of CHOP on HIV PI-induced inhibition of adipocyte differentiation. Primary murine pre-adipocytes were isolated from wild type (WT) and CHOP^−/−^ mice. Cells were induced to differentiate while concurrently treated with HIV PIs (12.5 µM) for 10 days. **A).** The intracellular lipid was stained with Oil Red O. Images were acquired with a 40× objective lens. Representative images for each treatment are shown. B–C) Bright field images were acquired with a 20×objective lens, and images were processed using MATLAB. The number and size of lipid droplets were measured. Values are mean ± SE for three independent experiments. Statistical significance relative to vehicle control, **p<0.01.

To identify the potential link between the UPR activation and alteration of intracellular lipid accumulation in adipocytes, we isolated primary adipocytes from C57BL/6 wild type and CHOP^−/−^ mice with a C57BL/6J background. Isolated primary adipocytes were induced to differentiate while concurrently treated with HIV PIs for 10 days. Intracellular lipid droplets were stained with Oil Red O. As shown in Figure 8A, similar to the findings in cultured murine and human adipocytes, LPV and LPV/RTV significantly inhibited intracellular lipid accumulation in wild-type mouse adipocytes. However, in the absence of CHOP, LPV and LPV/RTV had less effect on intracellular lipid accumulation. We further analyzed the effect of CHOP on LD formation using MATLAB. A shown in Figure 8B and C, HIV PI-induced inhibition of LD formation was abrogated in the absence of CHOP. These results suggest that HIV PI-induced CHOP expression contributes to the dysregulation of differentiation in adipocytes.

### Effect of HIV PIs on Autophagy Activation in Adipocytes

**Figure 9:**
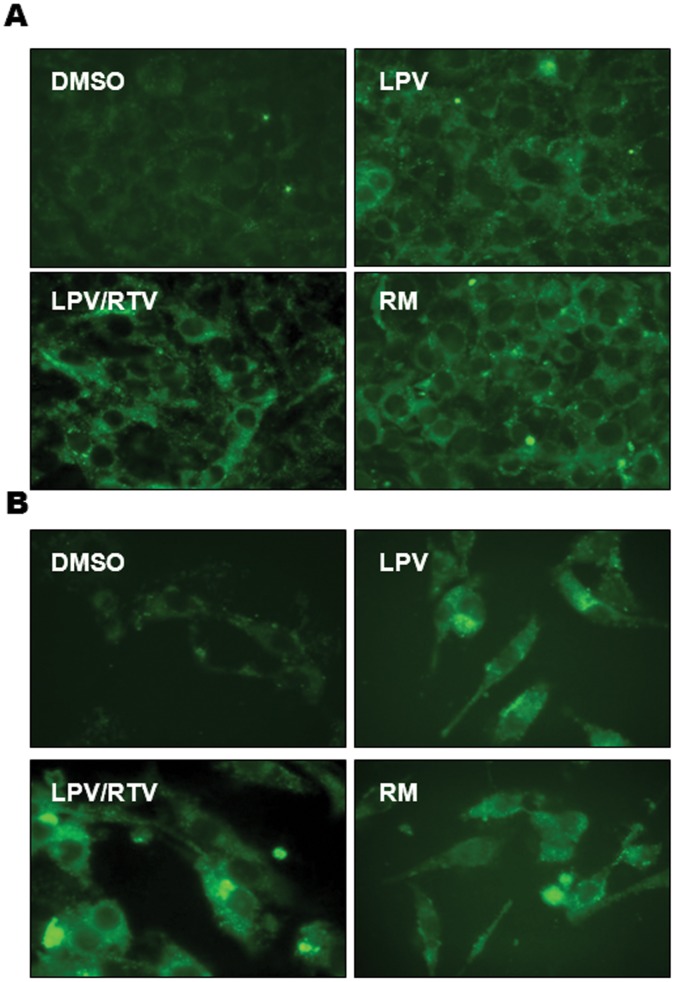
Effect of HIV PIs on autophagic punctate formation. 3T3-L1 cells stably expressing GFP-tagged LC3 were treated with different HIV PIs (12.5 µM), rapamycin (RM, 30 nM), or vehicle control (DMSO) for 24 h. The fluorescence images were recorded using a **A)** 40×, **B)** 63 × oil lens with FITC filter. Representative images for each treatment are shown.

**Figure 10:**
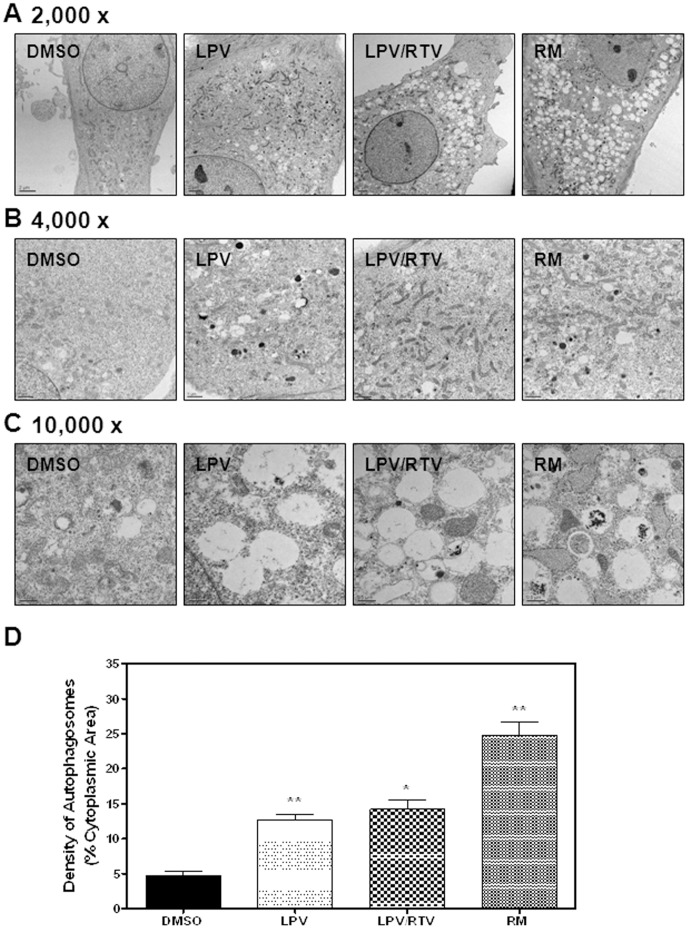
Effect of HIV PIs on autophagosome formation in 3T3-L1 cells. Non-differentiated 3T3-L1s were treated with individual HIV PIs (12.5 μM), rapamycin (RM, 30 nM) or vehicle control (DMSO) for 48 h. Cells were processed for transmission electron microscopy as described in **“**Methods”. **A–C)** Representative images for each treatment at 2,000 ×, 4,000 × or 10,000 × are shown. **C)** The density of autophagosomes for each treatment was point counted at 4,000 × and expressed as percentage of cytoplasmic area. Statistical significance relative to vehicle control: *p<0.05, **p<0.01.

Autophagy has been recently identified as a cellular target for dysregulation of lipid metabolism [45], and it regulates body lipid accumulation by controlling adipocyte differentiation [27]. Autophagy activity is also closely linked to ER stress signaling pathways [28], [46]. To investigate if autophagy dysfunction is involved in HIV PI-induced inhibition of adipocyte differentiation, non-differentiated 3T3-L1 cells stably transfected with GFP-LC3 were treated with HIV PIs (12.5 μM) or rapamycin (RM, 30 nM) for 24 h, the autophagic punctate dot formation was observed under fluorescence microscopy. As shown in Figure.9, LPV and LPV/RTV increased the number of autophagic punctate dots. We further confirmed the effect of HIV PIs on autophagosome accumulation using electron microscopy, a more accurate assessment of autophagy induction. As shown in Figure 10, there was a significant increase of autophagosome density with LPV and LPV/RTV in 3T3-L1 cells.

### Effect of HIV PIs on Autophagic Flux

**Figure 11:**
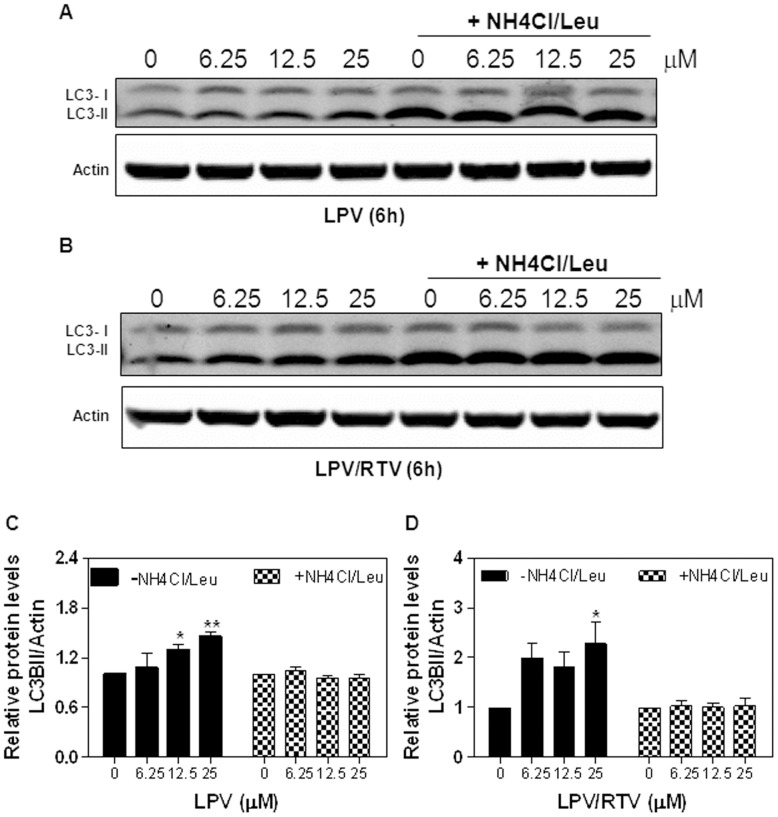
Effect of HIV PIs on autophagy flux in 3T3L1 cells. Non-differentiated 3T3-L1 cells were treated with different concentrations of HIV PIs for 6 h in the absence or presence of ammonium chloride (20 mM)/leupeptin (10 µM) (NH4Cl/Leu). Total cell lysates were isolated for Immunoblot analysis of LC3-I and LC3-II. β-actin was used as loading control. NH4Cl/Leu was added to the cells 2 h prior to harvesting. Representative immunoblots of **A**) LPV and **B**) LPV/RTV are shown. **C–D).** The density of immunoblot was determined by Image J. Relative a protein level of LC3-II was normalized with β-Actin. Values are mean ±SE of three independent experiments. Statistical significance relative to vehicle control, *p<0.05, **p<0.01.

The increase of autophagosome number does not always indicate the increase of autophagic activity. Accumulation of autophagosomes can be caused by an increase in the induction of autophagy or an impairment of autophagolysosomal maturation. We therefore examined the effect of HIV PIs on autophagic flux by measuring protein levels of membrane-associated form LC3-II in the presence of lysosome inhibitors (ammonium chloride/Leupetin) in 3T3L1 cells. As shown in Figure 11, although LPV and LPV/RTV increased the protein levels of LC3-II, in the presence of lysosome inhibitors, there was no further increase of LC3-II compared to DMSO control. Furthermore, HIV PIs had no effect on the expression of ATG5 and ATG7, which are two essential proteins involved in autophagosome formation (Supplementary Figure 6 in File S1). These results suggest that LPV and LPV/RTV may inhibit the degradation of LC3-II rather than induce autophagy activation.

**Figure 12:**
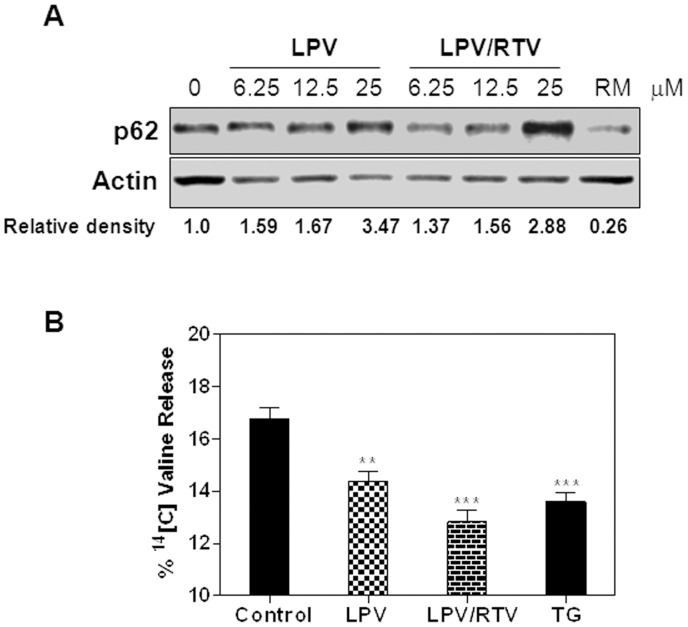
A) Effect of HIV PIs on p62 degradation. Differentiated 3T3-L1 cells were treated with different amounts of HIV PIs for 48 h, and total cell lysates were used to analyze p62 protein levels. Representative immunoblots of p62 and β-actin are shown. The density of the immunoblots was analyzed using Image J software. **B)** Effect of HIV PIs on long-lived protein degradation. Differentiated 3T3-L1 cells were metabolically labeled with ^14^C-valine, then treated with HIV PIs (12.5 µM) for 24 h. The degradation of long-lived protein was analyzed as described in “Methods”. Results are the mean ± SE of three independent experiments ***p<0.001.

To further determine the effect of HIV PIs on autophagic degradation, we examined the effect of LPV and LPV/RTV on the accumulation of p62. p62 is a nuclear membrane protein proposed to be specifically degraded through the autophagic pathway as it specifically binds to LC3-II and serves as a link to ubiquitinated substrates [47], [48]. Therefore, the activity of autophagy should inversely correlate with p62 protein levels. The results indicated that LPV and LPV/RTV dose-dependently increased the accumulation of p62 in differentiated 3T3-L1 adipocytes (Figure 12A), while RM significantly decreased p62 protein level. To further follow this observation, we examined the effect of HIV PIs on degradation of long-lived protein using ^14^C-valine labeled differentiated 3T3-L1 adipocytes. As shown in Figure 12B, LPV and LPV/RTV significantly inhibited autophagic proteolysis of long-lived proteins in adipocytes. TG, a known ER stress activator, also showed significant inhibition. Taken together, these results suggest that the degradation of autophagic components was inhibited by HIV protease inhibitors.

### Effect of HIV PIs on ER Calcium Stores in Adipocytes

**Figure 13:**
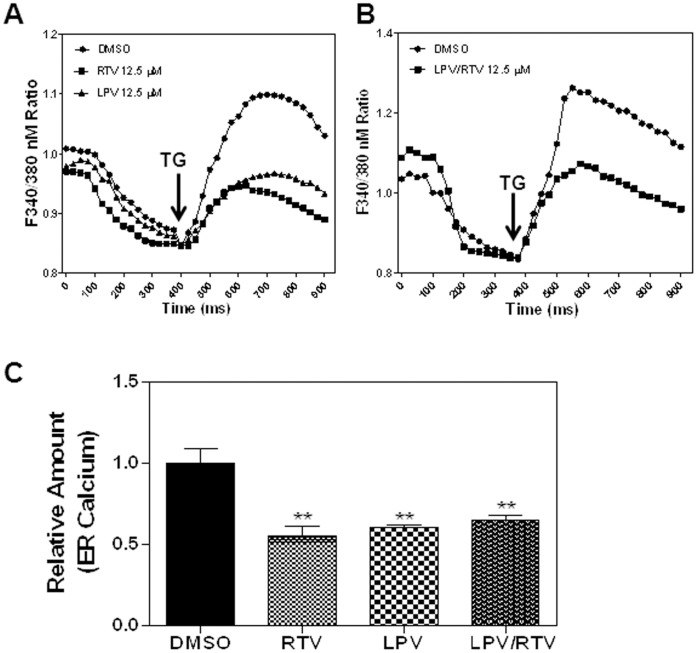
Effect of HIV PIs on ER calcium stores. Non-differentiated 3T3-L1 cells were treated with HIV PIs (RTV, LPV, or LPV/RTV, 12.5 μM) or vehicle control (DMSO) for 24 h. ER calcium stores were assessed by Fura-2 fluorescence ratio of 340∶380 nm in individual cells before and after addition of thapsigargin (TG, 100 nM). Representative tracing for a summation of at least 15 cells is shown. **A)** Cells were treated with RTV or LPV. **B)** Cells were treated with LPV/RTV. **C)** Relative calcium content was calculated by total area under the curve for each treatment group and compared to vehicle control (which was set as 1). Statistical significance relative to vehicle control, **p<0.01.

ER stress can be activated by a number of insults. Maintenance of ER calcium homeostasis is essential for many cellular functions. TG, a sarcoplasmic/ER calcium ATPase inhibitor, depletes the ER calcium stores and activates the UPR in many different cells [49], [50]. We have previously shown RTV depletes ER calcium in macrophages leading to UPR activation [3]. We further examined the effect of LPV, RTV and LPV/RTV on ER calcium stores in adipocytes. 3T3-L1 pre-adipocytes were treated with HIV PIs (12.5 μM) or vehicle control (DMSO) for 24 h, and the ER calcium content was determined using the fluorescent calcium indicator Fura-2/AM as described previously [3]. As shown in Figure 13, RTV-, LPV- or LPR/RTV-treated cells markedly reduced the response to TG, indicating that ER calcium stores were depleted. A recent study has reported that TG specifically blocks autophagosomal-lysosomal fusion and inhibits autophagy activity [51]. The HIV PI-induced ER calcium depletion may also account for the observed inhibition of autophagy activity.

## Discussion

Although HIV PIs are extremely effective in decreasing viral load, HIV patients on HAART containing HIV PIs are at increased risk for developing metabolic syndrome and cardiovascular diseases [36], [52]. The field of ER stress and UPR has gained great attention during the last decade. The UPR signaling pathway plays an important role in regulating normal functions of various cells including hepatocytes, β-cells, and macrophages. Consequently, dysregulation of the UPR signaling pathway has been implicated in various human diseases such as diabetes, fatty liver, and cardiovascular diseases [53]–[56].

An old paradigm of dyslipidemia considered hepatocytes and macrophages as central players in the lipid metabolism dysregulation. In parallel, investigations attempting to determine the cellular mechanism of HIV PI-induced dyslipidemia have thoroughly focused on these two cell types. However, increasing evidence demonstrates that adipose tissue is a central player in metabolic disease, including dysregulation of lipid metabolism and insulin resistance [14], [57], [58].

Through the current studies, we have attempted to elucidate the mechanistic dysregulations induced by HIV PIs in adipocytes. Previous studies from our laboratory suggest that activation of ER stress represents an important cellular mechanism underlying HIV PI-induced inflammation and dyslipidemia [3], [5], [40]. In addition, studies from other investigators have linked ER stress to HIV PI-induced insulin resistance and dysfunction in adipocytes [20], [36]. A recent study by Capel, *et al*, showed that LPV/RTV adversely affected differentiation, lipid content, mitochondrial function, ROS production and insulin sensitivity in adipocytes [37]. However, little is known of how HIV PIs affect lipid metabolism in adipocytes.

In the current study, we show that HIV PIs induce ER stress in a time and dose-dependent manner in adipocytes, similar to what has been shown in macrophages and hepatocytes (Figure 1–4 and Supplementary Figure 1–4 in File S1) [3], [40]. However, not all HIV PIs activated the UPR to the same extent, which may be explained by pharmacokinetic differences within this drug class. HIV PIs differ in molecular weight, ionization, and lipophilicity (oil-water partition coefficient, or log P), all of which determine rate of transport through cellular membranes [59], [60]. Interestingly, these differences of log P and cellular accumulation parallel our observations of ER stress activation i.e. high for NEV and SQV, intermediate for IDV, RTV and LPV, and lower for DAV [59], [61]–[65]. While further study is needed to conclusively determine the log P and intracellular concentrations for LPV and RTV, the connection is notable and may correlate to observed difference of dyslipidemia induction in the clinic.

In this study, we have focused on the most widely used HIV PIs, LPV/RTV (4∶1), which has been demonstrated to induce dyslipidemia in patients [66]–[68], and to study the correlation of ER stress activation and alterations in adipogenesis. LPV/RTV has previously been shown to induce metabolic alternations in various cells and tissues [3]–[5], [39], [69]. Our results indicated that LPV/RTV significantly activate the UPR and inhibit adipocyte differentiation in physiologic concentration ranges (6.25 to 25 μM) [70]. We hypothesized that the induction of ER stress may be the cause of these alterations, as the ER and LD organelles are so closely intertwined [71]–[74]. Indeed, knockout of CHOP, a downstream UPR transcription factor, abrogated HIV PI-induced dysregulation of adipogenesis. While we demonstrated this phenomenon in primary adipocytes in this particular study, we also have preliminary data indicating that this occurs *in vivo*.

It is now clear that autophagy also plays a critical role in lipid droplet formation and metabolism [75], [76]. Recent study by Gibellini *et al,* reported that AZV induced both apoptosis (high doses) and autophagy (low dose) in human cancer preadipocytes [76]. However, the concentration of AZV used in this study was at a non-physiological range (50–200 µM). The physiological concentration of AZV ranges from 10–15 μM [77]. In addition, increase of LC3-II and GFP-LC3 punctae formation may be caused by inhibition of autophagy flux rather than increased activity of the pathway.

Our current study further investigated the effect of HIV PIs at clinically relevant concentration on autophagy induction and activity in our model. We have demonstrated that LPV, LPV/RTV and RTV do induce autophagosome accumulation in murine adipocytes (Figure 9, 10 and supplementary Figure 5 in File S1). Recently, it has been reported that TG, an ER stress inducer widely used to induce autophagy, actually inhibited autophagy activity [51]. Our results also indicated that activation of the UPR is correlated to HIV PI-induced inhibition of autophagy activity and adipocyte differentiation. Previous studies have shown that HIV PIs also inhibit the activity of the 26S proteasome at high concentration, with LPV and RTV inhibiting chymotryptic activity 50–60% at 25 µM [78]. Therefore, our findings of p62 accumulation at high concentrations of LPV and LPV/RTV may be partially due to the inhibition of proteasome activity in addition to the inhibition of autophagy activity. Analysis of autophagic proteolysis of long-lived proteins further confirmed that HIV PIs inhibited autophagy flux. However, how HIV PI-induced ER stress and autophagy are entwined remains to be fully examined. Recent studies done by Yu *et al* identified CHOP as a regulator of macroautophagy. CHOP deficiency attenuated spinal and bulbar muscular atrophy, a disease triggered in part through ER stress activation *via* activation of macroautophagy [79]. Our recent studies also showed that HIV PI-induced inhibition of autophagy activity in primary mouse hepatocytes is also reversed in the absence of CHOP (unpublished data). Whether and how CHOP is involved in HIV PI-induced dysregulation of autophagy activity in adipocytes remains to be further identified and is our ongoing project.

In summary, we have shown that HIV PIs differentially activate ER stress in adipocytes, alter differentiation, disrupt the expression of key regulatory genes involved in lipid metabolism, and inhibit autophagy flux. These alterations are unlikely separate phenomenon, but rather intertwined with the activation of ER stress *via* depletion of ER calcium. Molecules that modulate ER stress response would be potential therapeutic targets for various diseases including HIV PI-induced dysregulation of lipid metabolism and metabolic syndrome.

## Supporting Information

File S1. (PDF)

## References

[1] Carr A, Cooper DA (1998) Images in clinical medicine. Lipodystrophy associated with an HIV-protease inhibitor. The New England journal of medicine 339: 1296. 9791146 10.1056/NEJM199810293391806

[2] Group DADS, Friis-Moller N, Reiss P, Sabin CA, Weber R, et al (2007) Class of antiretroviral drugs and the risk of myocardial infarction. The New England journal of medicine 356: 1723–1735. 17460226 10.1056/NEJMoa062744

[3] Zhou H, Pandak WM Jr, Lyall V, Natarajan R, Hylemon PB (2005) HIV protease inhibitors activate the unfolded protein response in macrophages: implication for atherosclerosis and cardiovascular disease. Molecular pharmacology 68: 690–700. 15976036 10.1124/mol.105.012898

[4] Zhou H, Gurley EC, Jarujaron S, Ding H, Fang Y, et al (2006) HIV protease inhibitors activate the unfolded protein response and disrupt lipid metabolism in primary hepatocytes. American journal of physiologyGastrointestinal and liver physiology 291: G1071–1080. 10.1152/ajpgi.00182.2006 16861219

[5] Wu X, Sun L, Zha W, Studer E, Gurley E, et al (2010) HIV protease inhibitors induce endoplasmic reticulum stress and disrupt barrier integrity in intestinal epithelial cells. Gastroenterology 138: 197–209. 19732776 10.1053/j.gastro.2009.08.054 PMC4644065

[6] Gills JJ, Lopiccolo J, Tsurutani J, Shoemaker RH, Best CJ, et al (2007) Nelfinavir, A lead HIV protease inhibitor, is a broad-spectrum, anticancer agent that induces endoplasmic reticulum stress, autophagy, and apoptosis in vitro and in vivo. Clin Cancer Res 13: 5183–5194. 17785575 10.1158/1078-0432.CCR-07-0161

[7] McLean K, VanDeVen NA, Sorenson DR, Daudi S, Liu JR (2009) The HIV protease inhibitor saquinavir induces endoplasmic reticulum stress, autophagy, and apoptosis in ovarian cancer cells. Gynecol Oncol 112: 623–630. 19147209 10.1016/j.ygyno.2008.11.028

[8] Fiorenza CG, Chou SH, Mantzoros CS (2011) Lipodystrophy: pathophysiology and advances in treatment. Nat Rev Endocrinol 7: 137–150. 21079616 10.1038/nrendo.2010.199 PMC3150735

[9] Lionetti L, Mollica MP, Lombardi A, Cavaliere G, Gifuni G, et al (2009) From chronic overnutrition to insulin resistance: the role of fat-storing capacity and inflammation. Nutrition, metabolism, and cardiovascular diseases : NMCD 19: 146–152. 10.1016/j.numecd.2008.10.010 19171470

[10] Bays HE, Gonzalez-Campoy JM, Henry RR, Bergman DA, Kitabchi AE, et al (2008) Is adiposopathy (sick fat) an endocrine disease? Int J Clin Pract 62: 1474–1483. 18681905 10.1111/j.1742-1241.2008.01848.x PMC2658008

[11] Virtue S, Vidal-Puig A (2010) Adipose tissue expandability, lipotoxicity and the Metabolic Syndrome–an allostatic perspective. Biochim Biophys Acta 1801: 338–349. 20056169 10.1016/j.bbalip.2009.12.006

[12] Belfort R, Berria R, Cornell J, Cusi K (2010) Fenofibrate reduces systemic inflammation markers independent of its effects on lipid and glucose metabolism in patients with the metabolic syndrome. The Journal of clinical endocrinology and metabolism 95: 829–836. 20061429 10.1210/jc.2009-1487 PMC2840858

[13] Bobbert T, Weicht J, Mai K, Mohlig M, Pfeiffer AF, et al (2009) Acute hyperinsulinaemia and hyperlipidaemia modify circulating adiponectin and its oligomers. Clinical endocrinology 71: 507–511. 19751297 10.1111/j.1365-2265.2008.03519.x

[14] Rasouli N, Kern PA (2008) Adipocytokines and the metabolic complications of obesity. The Journal of clinical endocrinology and metabolism 93: S64–73. 18987272 10.1210/jc.2008-1613 PMC2585759

[15] Cianflone K, Zakarian R, Stanculescu C, Germinario R (2006) Protease inhibitor effects on triglyceride synthesis and adipokine secretion in human omental and subcutaneous adipose tissue. Antivir Ther 11: 681–691. 17310812

[16] Lagathu C, Eustace B, Prot M, Frantz D, Gu Y, et al (2007) Some HIV antiretrovirals increase oxidative stress and alter chemokine, cytokine or adiponectin production in human adipocytes and macrophages. Antivir Ther 12: 489–500. 17668557

[17] Lagathu C, Kim M, Maachi M, Vigouroux C, Cervera P, et al (2005) HIV antiretroviral treatment alters adipokine expression and insulin sensitivity of adipose tissue in vitro and in vivo. Biochimie 87: 65–71. 15733739 10.1016/j.biochi.2004.12.007

[18] Giralt M, Domingo P, Villarroya F (2011) Adipose tissue biology and HIV-infection. Best Pract Res Clin Endocrinol Metab 25: 487–499. 21663842 10.1016/j.beem.2010.12.001

[19] Guallar JP, Gallego-Escuredo JM, Domingo JC, Alegre M, Fontdevila J, et al (2008) Differential gene expression indicates that ‘buffalo hump’ is a distinct adipose tissue disturbance in HIV-1-associated lipodystrophy. Aids 22: 575–584. 18316998 10.1097/QAD.0b013e3282f56b40

[20] Djedaini M, Peraldi P, Drici MD, Darini C, Saint-Marc P, et al (2009) Lopinavir co-induces insulin resistance and ER stress in human adipocytes. Biochemical and biophysical research communications 386: 96–100. 19501568 10.1016/j.bbrc.2009.05.148

[21] Gagnon A, Angel JB, Sorisky A (1998) Protease inhibitors and adipocyte differentiation in cell culture. Lancet 352: 1032. 9759749 10.1016/S0140-6736(05)60074-8

[22] Jones SP, Janneh O, Back DJ, Pirmohamed M (2005) Altered adipokine response in murine 3T3-F442A adipocytes treated with protease inhibitors and nucleoside reverse transcriptase inhibitors. Antiviral Therapy 10: 207–213. 15865214

[23] Jones SP, Waitt C, Sutton R, Back DJ, Pirmohamed M (2008) Effect of atazanavir and ritonavir on the differentiation and adipokine secretion of human subcutaneous and omental preadipocytes. AIDS (London, England) 22: 1293–1298. 10.1097/QAD.0b013e3283021a4f 18580608

[24] Vernochet C, Azoulay S, Duval D, Guedj R, Cottrez F, et al (2005) Human immunodeficiency virus protease inhibitors accumulate into cultured human adipocytes and alter expression of adipocytokines. The Journal of biological chemistry 280: 2238–2243. 15525648 10.1074/jbc.M408687200

[25] Czaja MJ (2011) Autophagy in health and disease. 2. Regulation of lipid metabolism and storage by autophagy: pathophysiological implications. Am J Physiol Cell Physiol 298: C973–978. 10.1152/ajpcell.00527.2009 PMC2867392 20089934

[26] Yorimitsu T, Klionsky DJ (2007) Eating the endoplasmic reticulum: quality control by autophagy. Trends in cell biology 17: 279–285. 17481899 10.1016/j.tcb.2007.04.005

[27] Singh R, Kaushik S, Wang Y, Xiang Y, Novak I, et al (2009) Autophagy regulates lipid metabolism. Nature 458: 1131–1135. 19339967 10.1038/nature07976 PMC2676208

[28] Zhou L, Liu F (2011) Autophagy: roles in obesity-induced ER stress and adiponectin downregulation in adipocytes. Autophagy 6: 1196–1197. 10.4161/auto.6.8.13478 PMC3039722 20864818

[29] Wabitsch M, Brenner RE, Melzner I, Braun M, Moller P, et al (2001) Characterization of a human preadipocyte cell strain with high capacity for adipose differentiation. Int J Obes Relat Metab Disord 25: 8–15. 11244452 10.1038/sj.ijo.0801520

[30] Zhou H, Jarujaron S, Gurley EC, Chen L, Ding H, et al (2007) HIV protease inhibitors increase TNF-alpha and IL-6 expression in macrophages: involvement of the RNA-binding protein HuR. Atherosclerosis 195: e134–143. 17531241 10.1016/j.atherosclerosis.2007.04.008

[31] Greenspan P, Mayer EP, Fowler SD (1985) Nile red: a selective fluorescent stain for intracellular lipid droplets. J Cell Biol 100: 965–973. 3972906 10.1083/jcb.100.3.965 PMC2113505

[32] Zhou J, Lhotak S, Hilditch BA, Austin RC (2005) Activation of the unfolded protein response occurs at all stages of atherosclerotic lesion development in apolipoprotein E-deficient mice. Circulation 111: 1814–1821. 15809369 10.1161/01.CIR.0000160864.31351.C1

[33] Or-Tzadikario S, Sopher R, Gefen A (2010) Quantitative monitoring of lipid accumulation over time in cultured adipocytes as function of culture conditions: toward controlled adipose tissue engineering. Tissue Eng Part C Methods 16: 1167–1181. 20163242 10.1089/ten.TEC.2009.0755

[34] Komiya K, Uchida T, Ueno T, Koike M, Abe H, et al (2010) Free fatty acids stimulate autophagy in pancreatic beta-cells via JNK pathway. Biochem Biophys Res Commun 401: 561–567. 20888798 10.1016/j.bbrc.2010.09.101

[35] Roberts EA, Deretic V (2008) Autophagic proteolysis of long-lived proteins in nonliver cells. Methods Mol Biol 445: 111–117. 18425445 10.1007/978-1-59745-157-4_6

[36] Grigem S, Fischer-Posovszky P, Debatin KM, Loizon E, Vidal H, et al (2005) The effect of the HIV protease inhibitor ritonavir on proliferation, differentiation, lipogenesis, gene expression and apoptosis of human preadipocytes and adipocytes. Hormone and metabolic research 37: 602–609. 16278782 10.1055/s-2005-870526

[37] Capel E, Auclair M, Caron-Debarle M, Capeau J (2012) Effects of ritonavir-boosted darunavir, atazanavir and lopinavir on adipose functions and insulin sensitivity in murine and human adipocytes. Antivir Ther 17: 549–556. 22293506 10.3851/IMP1988

[38] Gallego-Escuredo JM, Del Mar Gutierrez M, Diaz-Delfin J, Domingo JC, Mateo MG, et al (2010) Differential effects of efavirenz and lopinavir/ritonavir on human adipocyte differentiation, gene expression and release of adipokines and pro-inflammatory cytokines. Curr HIV Res 8: 545–553. 21073442 10.2174/157016210793499222

[39] Noor MA, Flint OP, Maa JF, Parker RA (2006) Effects of atazanavir/ritonavir and lopinavir/ritonavir on glucose uptake and insulin sensitivity: demonstrable differences in vitro and clinically. AIDS 20: 1813–1821. 16954722 10.1097/01.aids.0000244200.11006.55

[40] Zhou H, Pandak WM Jr, Hylemon PB (2006) Cellular mechanisms of lipodystrophy induction by HIV protease inhibitors. Future Lipidology 1: 163.

[41] Williams K, Rao YP, Natarajan R, Pandak WM, Hylemon PB (2004) Indinavir alters sterol and fatty acid homeostatic mechanisms in primary rat hepatocytes by increasing levels of activated sterol regulatory element-binding proteins and decreasing cholesterol 7alpha-hydroxylase mRNA levels. Biochemical pharmacology 67: 255–267. 14698038 10.1016/j.bcp.2003.08.044

[42] Estrada V, Fuster M (2008) Darunavir in treatment-naive patients. The ARTEMIS study]. Enfermedades infecciosas y microbiologia clinica 26 Suppl 1010–13. 10.1016/s0213-005x(08)76548-0 19195454

[43] Lenhard JM, Furfine ES, Jain RG, Ittoop O, Orband-Miller LA, et al (2000) HIV protease inhibitors block adipogenesis and increase lipolysis in vitro. Antiviral Research 47: 121–129. 10996400 10.1016/s0166-3542(00)00102-9

[44] Pacenti M, Barzon L, Favaretto F, Fincati K, Romano S, et al (2006) Microarray analysis during adipogenesis identifies new genes altered by antiretroviral drugs. AIDS (London, England) 20: 1691–1705. 10.1097/01.aids.0000242815.80462.5a 16931933

[45] Dong H, Czaja MJ (2011) Regulation of lipid droplets by autophagy. Trends Endocrinol Metab 22: 234–240. 21419642 10.1016/j.tem.2011.02.003 PMC3118855

[46] Ogata M, Hino S, Saito A, Morikawa K, Kondo S, et al (2006) Autophagy is activated for cell survival after endoplasmic reticulum stress. Mol Cell Biol 26: 9220–9231. 17030611 10.1128/MCB.01453-06 PMC1698520

[47] Bjorkoy G, Lamark T, Pankiv S, Overvatn A, Brech A, et al (2009) Monitoring autophagic degradation of p62/SQSTM1. Methods Enzymol 452: 181–197. 19200883 10.1016/S0076-6879(08)03612-4

[48] Pankiv S, Clausen TH, Lamark T, Brech A, Bruun JA, et al (2007) p62/SQSTM1 binds directly to Atg8/LC3 to facilitate degradation of ubiquitinated protein aggregates by autophagy. J Biol Chem 282: 24131–24145. 17580304 10.1074/jbc.M702824200

[49] Goudeau H, Goudeau M (1998) Depletion of intracellular Ca2+ stores, mediated by Mg2+-stimulated InsP3 liberation or thapsigargin, induces a capacitative Ca2+ influx in prawn oocytes. Dev Biol 193: 225–238. 9473326 10.1006/dbio.1997.8799

[50] Torres M, Castillo K, Armisen R, Stutzin A, Soto C, et al (2011) Prion protein misfolding affects calcium homeostasis and sensitizes cells to endoplasmic reticulum stress. PLoS One 5: e15658. 10.1371/journal.pone.0015658 PMC3012133 21209925

[51] Ganley IG, Wong PM, Gammoh N, Jiang X (2011) Distinct autophagosomal-lysosomal fusion mechanism revealed by thapsigargin-induced autophagy arrest. Mol Cell 42: 731–743. 21700220 10.1016/j.molcel.2011.04.024 PMC3124681

[52] Flint OP, Noor MA, Hruz PW, Hylemon PB, Yarasheski K, et al (2009) The role of protease inhibitors in the pathogenesis of HIV-associated lipodystrophy: cellular mechanisms and clinical implications. Toxicol Pathol 37: 65–77. 19171928 10.1177/0192623308327119 PMC3170409

[53] Kaplowitz N, Than TA, Shinohara M, Ji C (2007) Endoplasmic reticulum stress and liver injury. Semin Liver Dis 27: 367–377. 17979073 10.1055/s-2007-991513

[54] Toth A, Nickson P, Mandl A, Bannister ML, Toth K, et al (2007) Endoplasmic reticulum stress as a novel therapeutic target in heart diseases. Cardiovasc Hematol Disord Drug Targets 7: 205–218. 17896961 10.2174/187152907781745260

[55] Lin JH, Walter P, Yen TS (2007) Endoplasmic Reticulum Stress in Disease Pathogenesis. Annu Rev Pathol. 3: 399–425. 10.1146/annurev.pathmechdis.3.121806.151434 PMC3653419 18039139

[56] Yoshida H (2007) ER stress and diseases. FEBS J 274: 630–658. 17288551 10.1111/j.1742-4658.2007.05639.x

[57] Ahima RS, Flier JS (2000) Adipose tissue as an endocrine organ. Trends in endocrinology and metabolism: TEM 11: 327–332. 10996528 10.1016/s1043-2760(00)00301-5

[58] Gustafson B (2010) Adipose tissue, inflammation and atherosclerosis. J Atheroscler Thromb 17: 332–341. 20124732 10.5551/jat.3939

[59] Ford J, Khoo SH, Back DJ (2004) The intracellular pharmacology of antiretroviral protease inhibitors. J Antimicrob Chemother 54: 982–990. 15537695 10.1093/jac/dkh487

[60] Zha W, Zha BS, Zhou F, Zhou H, Wang G (2012) The Cellular Pharmacokinetics of HIV Protease Inhibitors: Current Knowledge and Future Perspectives. Curr Drug Metab 13: 1174–1183. 22746305 10.2174/138920012802850119

[61] Elens L, Yombi JC, Lison D, Wallemacq P, Vandercam B, et al (2009) Association between ABCC2 polymorphism and lopinavir accumulation in peripheral blood mononuclear cells of HIV-infected patients. Pharmacogenomics 10: 1589–1597. 19842932 10.2217/pgs.09.88

[62] Fayet Mello A, Buclin T, Franc C, Colombo S, Cruchon S, et al (2011) Cell disposition of raltegravir and newer antiretrovirals in HIV-infected patients: high inter-individual variability in raltegravir cellular penetration. J Antimicrob Chemother 66: 1573–1581. 21508009 10.1093/jac/dkr151

[63] Hennessy M, Clarke S, Spiers JP, Kelleher D, Mulcahy F, et al (2004) Intracellular accumulation of nelfinavir and its relationship to P-glycoprotein expression and function in HIV-infected patients. Antivir Ther 9: 115–122. 15040543

[64] Hennessy M, Clarke S, Spiers JP, Mulcahy F, Kelleher D, et al (2003) Intracellular indinavir pharmacokinetics in HIV-infected patients: comparison with plasma pharmacokinetics. Antivir Ther 8: 191–198. 12924535

[65] Meaden ER, Hoggard PG, Newton P, Tjia JF, Aldam D, et al (2002) P-glycoprotein and MRP1 expression and reduced ritonavir and saquinavir accumulation in HIV-infected individuals. J Antimicrob Chemother 50: 583–588. 12356805 10.1093/jac/dkf161

[66] Calza L, Manfredi R, Pocaterra D, Chiodo F (2008) Efficacy and tolerability of a fosamprenavir-ritonavir-based versus a lopinavir-ritonavir-based antiretroviral treatment in 82 therapy-naive patients with HIV-1 infection. Int J STD AIDS 19: 541–544. 18663041 10.1258/ijsa.2008.007322

[67] Lafeuillade A, Hittinger G, Philip G, Lambry V, Jolly P, et al (2004) Metabolic evaluation of HIV-infected patients receiving a regimen containing lopinavir/ritonavir (Kaletra). HIV Clin Trials 5: 392–398. 15682352 10.1310/Q0TG-0V50-9JML-638U

[68] Rhee MS, Hellinger JA, Sheble-Hall S, Cohen CJ, Greenblatt DJ (2010) Relationship between plasma protease inhibitor concentrations and lipid elevations in HIV patients on a double-boosted protease inhibitor regimen (saquinavir/lopinavir/ritonavir). J Clin Pharmacol 50: 392–400. 20097936 10.1177/0091270009339739

[69] Wang Y, Zhang L, Wu X, Gurley EC, Kennedy E, et al.. (2012) The role of C/EBP homologous protein in HIV protease inhibitor-induced hepatic lipotoxicity. Hepatology. 10.1002/hep.26107 PMC3566321 23080229

[70] Jackson A, Hill A, Puls R, Else L, Amin J, et al (2011) Pharmacokinetics of plasma lopinavir/ritonavir following the administration of 400/100 mg, 200/150 mg and 200/50 mg twice daily in HIV-negative volunteers. J Antimicrob Chemother 66: 635–640. 21172791 10.1093/jac/dkq468 PMC3594886

[71] Robenek H BI, Robenek MJ, Hofnagel O, Ruebel A, Troyer D, Severs NJ (2010) Topography of lipid droplet-associated proteins: insights from freeze-fracture replica immunogold labeling. Journal of Lipids 2011: 1–10. 10.1155/2011/409371 PMC3068475 21490801

[72] Walther TC, Farese RV Jr (2009) The life of lipid droplets. Biochim Biophys Acta 1791: 459–466. 19041421 10.1016/j.bbalip.2008.10.009 PMC2782899

[73] Sriburi R, Bommiasamy H, Buldak GL, Robbins GR, Frank M, et al (2007) Coordinate regulation of phospholipid biosynthesis and secretory pathway gene expression in XBP-1(S)-induced endoplasmic reticulum biogenesis. J Biol Chem 282: 7024–7034. 17213183 10.1074/jbc.M609490200

[74] Sriburi R, Jackowski S, Mori K, Brewer JW (2004) XBP1: a link between the unfolded protein response, lipid biosynthesis, and biogenesis of the endoplasmic reticulum. J Cell Biol 167: 35–41. 15466483 10.1083/jcb.200406136 PMC2172532

[75] Singh R, Xiang Y, Wang Y, Baikati K, Cuervo AM, et al (2009) Autophagy regulates adipose mass and differentiation in mice. The Journal of clinical investigation 119: 3329–3339. 19855132 10.1172/JCI39228 PMC2769174

[76] Gibellini L, De Biasi S, Pinti M, Nasi M, Riccio M, et al (2012) The protease inhibitor atazanavir triggers autophagy and mitophagy in human preadipocytes. Aids 26: 2017–2026. 22948272 10.1097/QAD.0b013e328359b8be

[77] Zhu L, Butterton J, Persson A, Stonier M, Comisar W, et al (2010) Pharmacokinetics and safety of twice-daily atazanavir 300 mg and raltegravir 400 mg in healthy individuals. Antivir Ther 15: 1107–1114. 21149917 10.3851/IMP1673

[78] Parker RA, Flint OP, Mulvey R, Elosua C, Wang F, et al (2005) Endoplasmic reticulum stress links dyslipidemia to inhibition of proteasome activity and glucose transport by HIV protease inhibitors. Mol Pharmacol 67: 1909–1919. 15755908 10.1124/mol.104.010165

[79] Yu Z, Wang AM, Adachi H, Katsuno M, Sobue G, et al (2011) Macroautophagy is regulated by the UPR-mediator CHOP and accentuates the phenotype of SBMA mice. PLoS Genet 7: e1002321. 22022281 10.1371/journal.pgen.1002321 PMC3192827

