# Supplementary material for: HIV Protease Inhibitors Disrupt Lipid Metabolism by Activating Endoplasmic Reticulum Stress and Inhibiting Autophagy Activity in Adipocytes
Source: PLoS One. 2013 Mar 22;8(3):e59514. doi: 10.1371/journal.pone.0059514 (PMC3606318; doi:10.1371/journal.pone.0059514)
Supplement: File S1. — (PDF) [file pone.0059514.s001.pdf]

Supplementary Figure 1

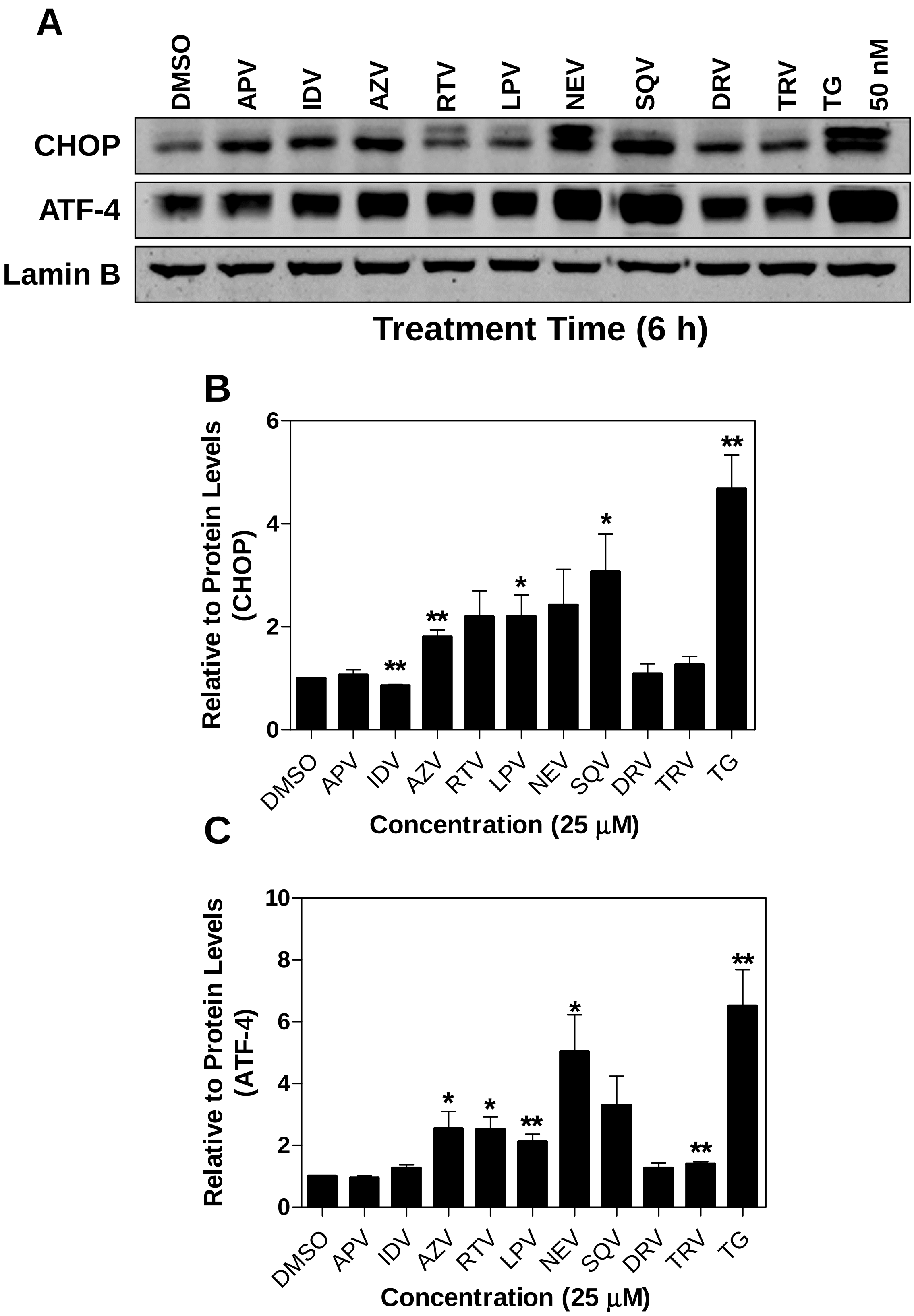

Supplementary Figure 2

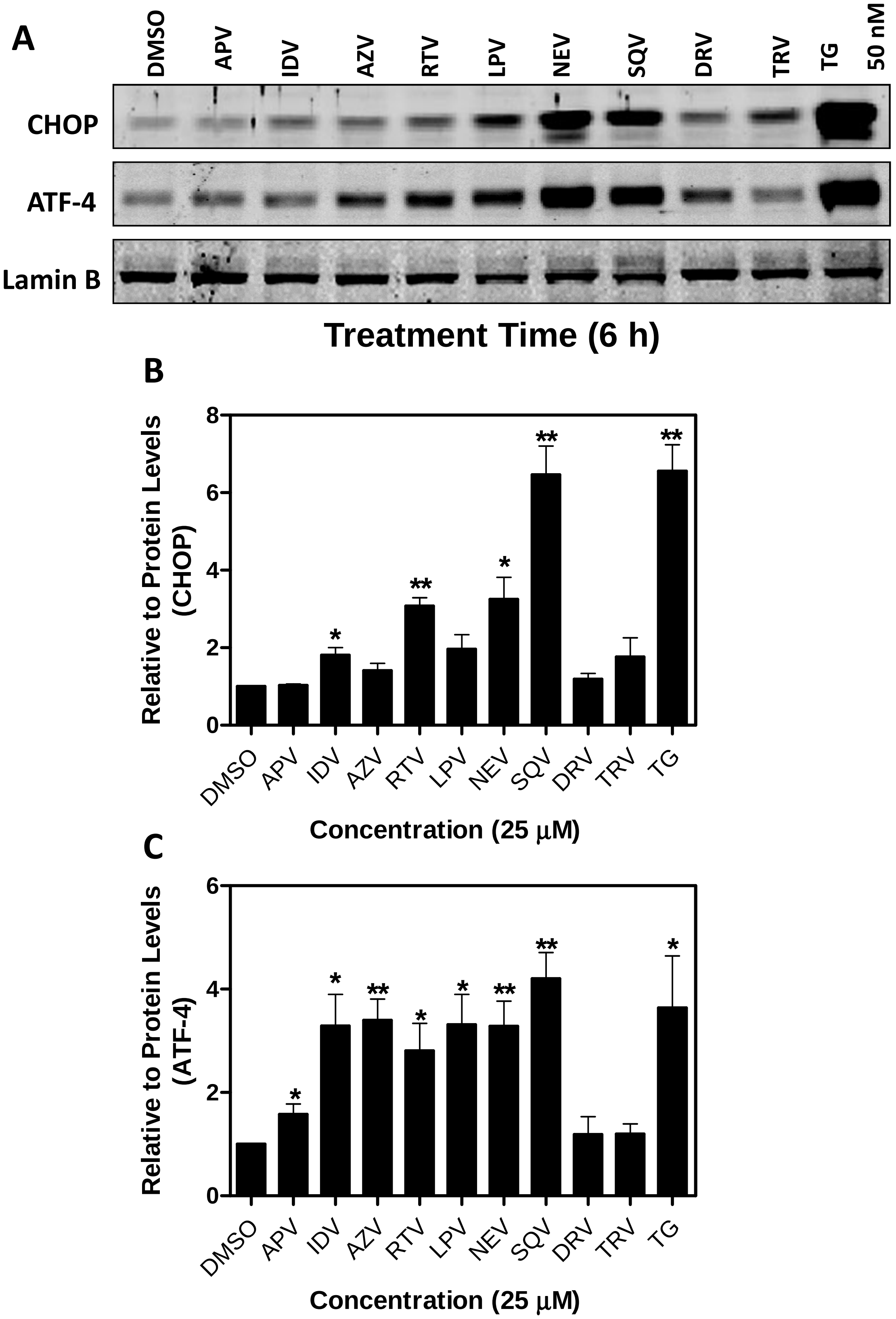

Supplementary Figure 3

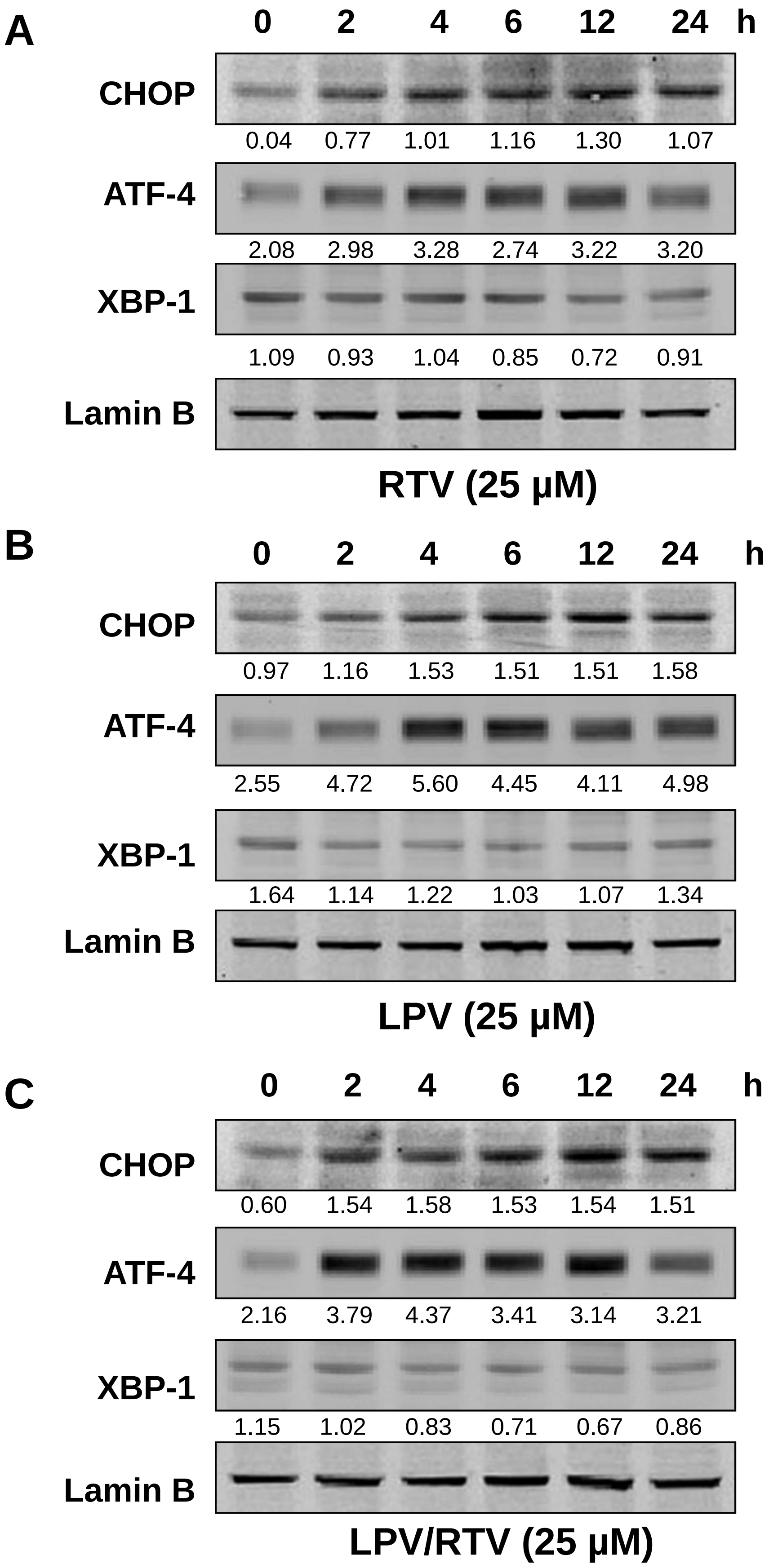

Supplementary Figure 4

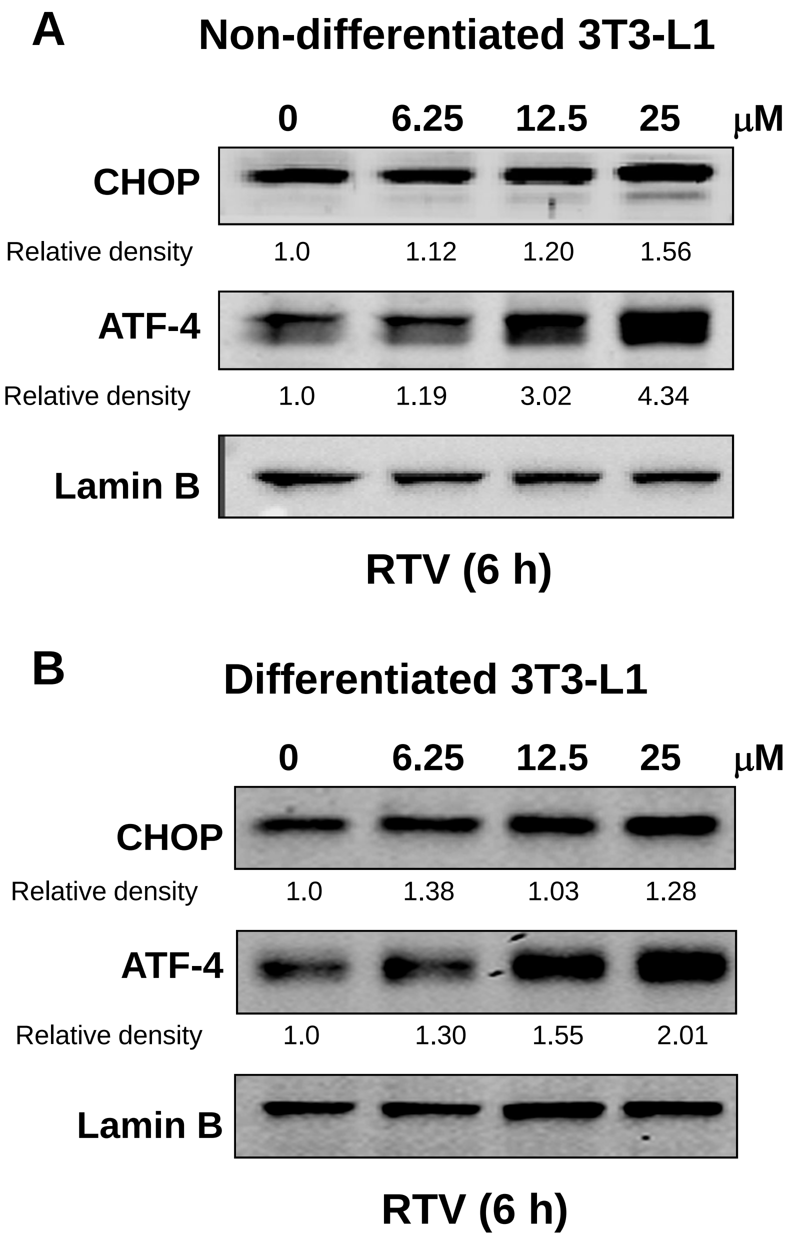

Supplementary Figure 4

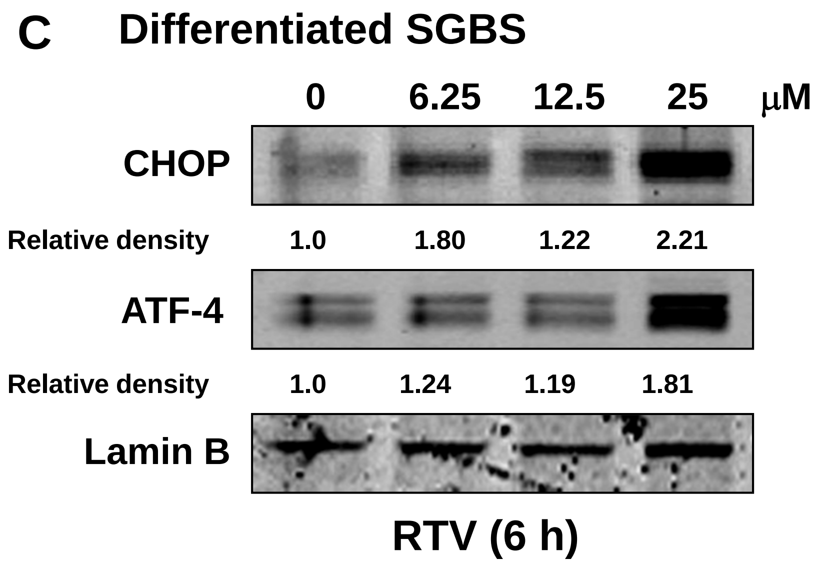

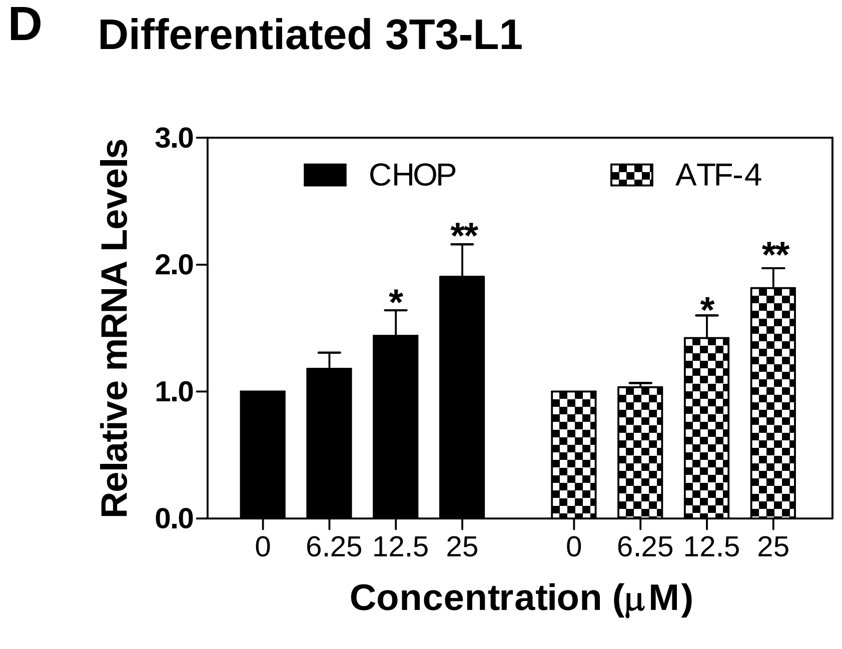

Supplementary Figure 5

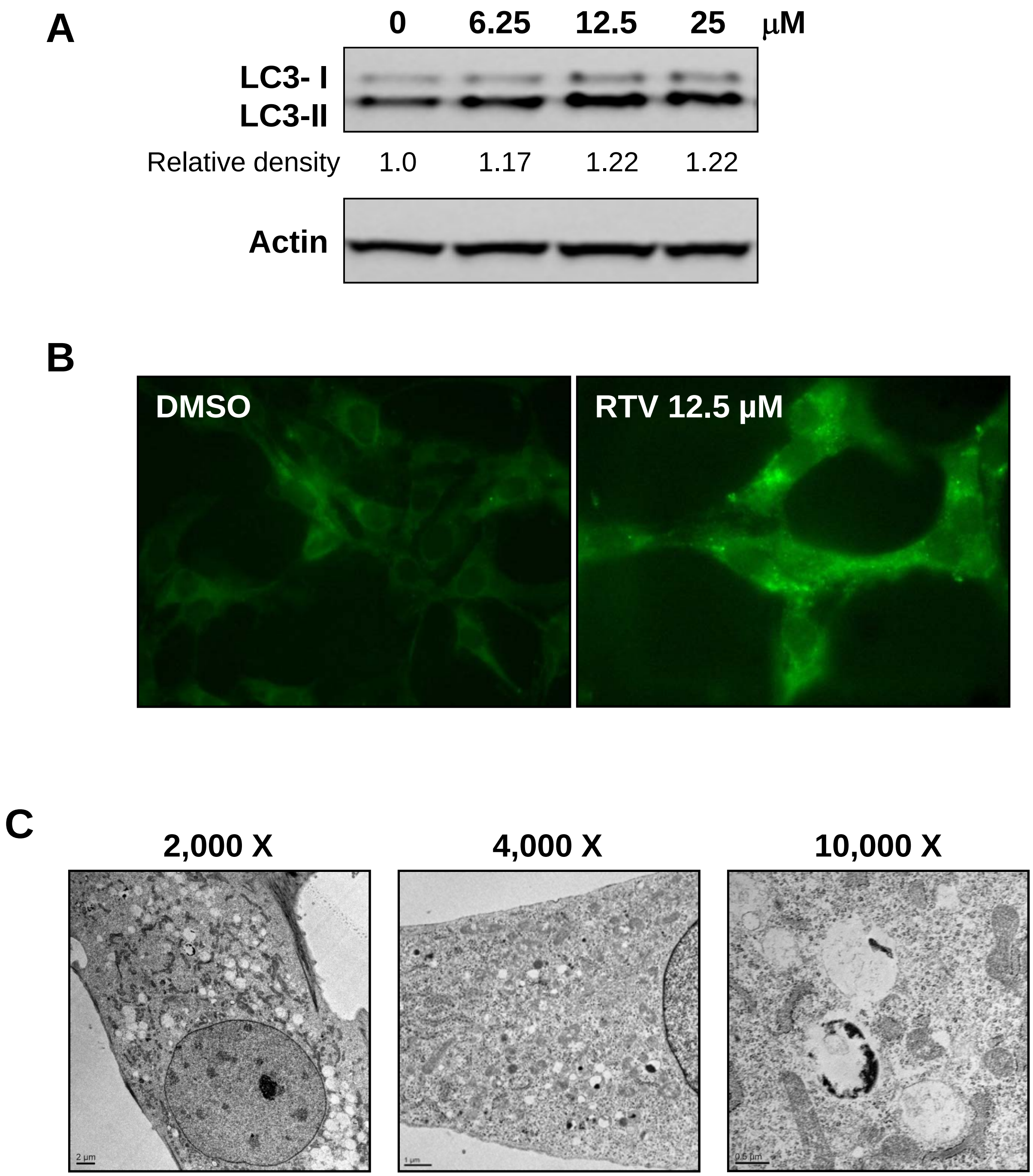

Supplementary Figure 6

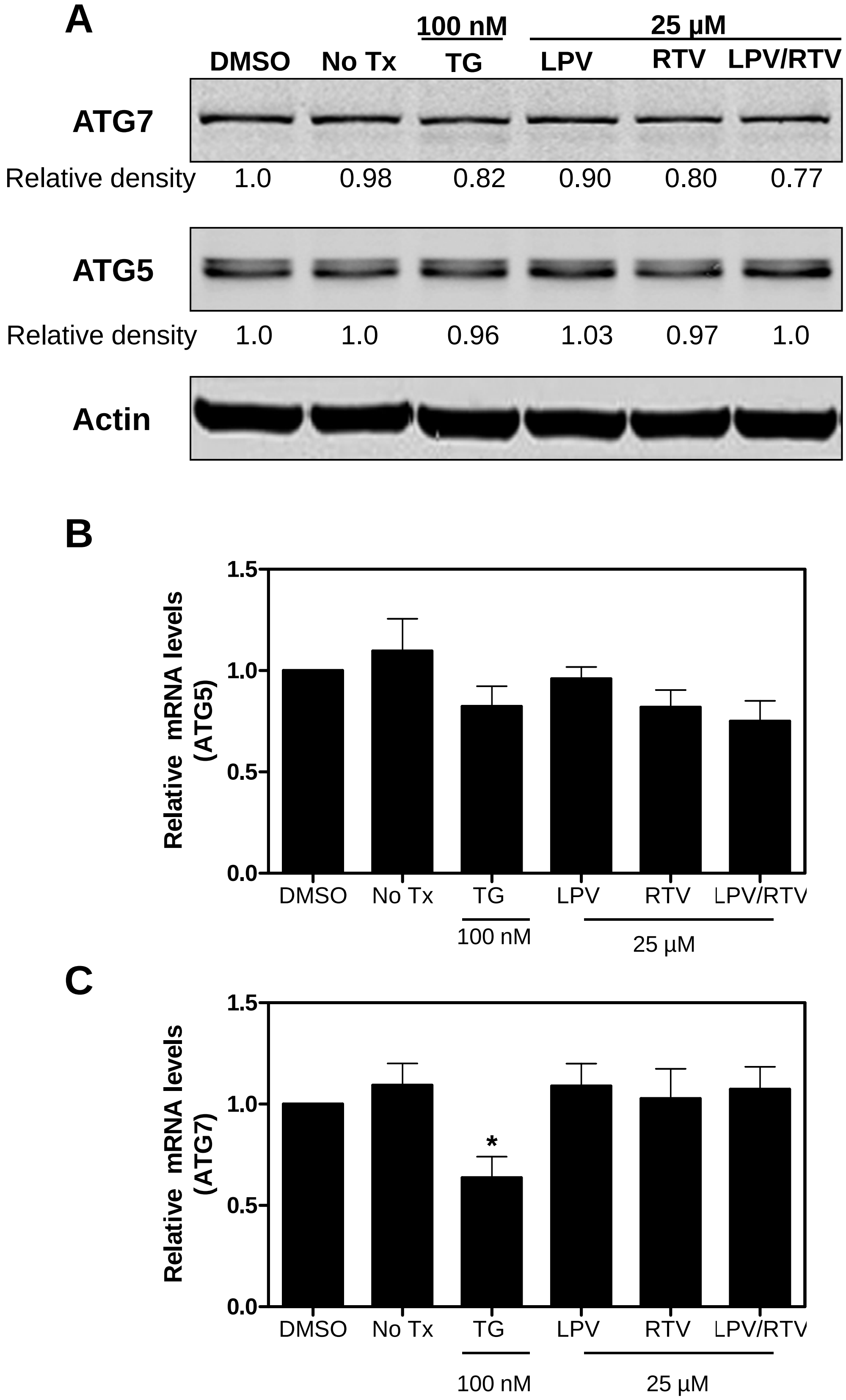

Supplementary Figure 1. Differential activation of the UPR by HIV PIs in non-differentiated 3T3-L1 cells. Non-differentiated 3T3-L1 cells were treated for 6 hwith 25 μM of 9 HIV PIs. Amprenavir (APV), Indinavir (IDV), Atazanavir (AZV), Ritonavir (RTV), Lopinavir (LPV), Nelfinavir (NEV), Saquinavir (SQV), Darunavir (DAV), Tipranavir (TRV). **A)** Representative immunoblots against CHOP, ATF-4 and Lamin B from nuclear extracts are shown. **B-C)** The density of immunoblots was determined by Image J. Relative protein levels of CHOP and ATF-4 were normalized using Lamin B as loading control. Values are mean ± SE of three independent experiments. Statistical significance relative to vehicle control, *p < 0.05 and **p<0.01.

Supplementary Figure 2. Differential activation of the UPR by HIV PIs in differentiated 3T3-L1 cells. Non-differentiated 3T3-L1 cells were treated for 6 h with 25 μM of 9 HIV PIs. Amprenavir (APV), Indinavir (IDV), Atazanavir (AZV), Ritonavir (RTV), Lopinavir (LPV), Nelfinavir (NEV), Saquinavir (SQV), Darunavir (DAV), Tipranavir (TRV). **A)** Representative immunoblots against CHOP, ATF-4 and Lamin B from nuclear extracts. **B-C)** The density of immunoblots was determined by Image J. Relative protein levels of CHOP and ATF-4 were normalized using Lamin B as loading control. Values are mean ± SE of three independent experiments. Statistical significance relative to vehicle control, *p < 0.05 and **p<0.01.

Supplementary Figure 3. Time-dependent activation of the UPR in mouse adipocytes by HIV PIs. Representative immunoblots of five separate experiments against CHOP, ATF-4, XBP-1, and Lamin B from the nuclear extracts of differentiated mouse 3T3-L1 cells treated with 25 μM of HIV PIs for 0-24 h. Lamin B was used as loading control. **A)** Ritonavir (RTV); **B)** Lopinavir (LPV); **C)** Lopinavir/Ritonavir (LPV/RTV). The density of immunoblots was determined by Image J. Relative protein levels of CHOP and ATF-4 were normalized using Lamin B as loading control.

Supplementary Figure 4. Activation of the UPR by RTV. A) Non-differentiated 3T3L1 cells, **B)** differentiated 3T3-L1 cells, and **C)** differentiated human SGBS were treated with increasing concentrations of RTV for 6 h. Representative immunoblots against CHOP and ATF-4 from nuclear extracts are shown. The density of immunoblots was determined by Image J. Relative protein levels of CHOP and ATF-4 were normalized using Lamin B as a loading control. **D)** Differentiated 3T3-L1 cells were treated with increasing concentrations of RTV for 4 h and total RNA was isolated. The mRNA levels of CHOP and ATF-4 were quantified by real-time RT-PCR and normalized using internal control β-Actin. Values are mean ± SE of three independent experiments. Statistical significance relative to vehicle control, $*p < 0.05$.

Supplementary Figure 5. RTV induces an increase of autophagosomes in 3T3-L1 cells. A) Representative immunoblots of LC3 from total cell lysates of differentiated 3T3-L1 cells treated with increasing concentrations of RTV for 24 h are shown. β-actin was used as loading control. **B)** Representative fluorescent images of non-differentiated 3T3-L1 cells stably expressing GFP-tagged LC3 treated with 12.5 μM RTV or vehicle control (DMSO) for 24 h. **C)** Representative EM images of non-differentiated 3T3-L1s treated with individual 12.5 μM RTV for 24 h. Cells were processed for transmission electron microscopy as described in **"**Methods**".** Representative images at 2,000 ×, 4,000 × and 10,000 × are shown.

Supplemental Figure 6. Effect of HIV PIs on ATG5 and ATG7 expression in differentiated mouse adipocytes. A) Representative immunoblots against ATG7 and ATG5 from total cellular extracts of mouse differentiated 3T3-L1 cells treated with vehicle control (DMSO), TG 100 nM, or LPV (25 μM), RTV (25 μM), or LPV/RTV (4:1, LPV=20 μM, RTV=5μM), for 24 h are shown. The density of immunoblot was determined by Image J. Relative protein levels of ATG5 and ATG7 were normalized using β-Actin as a loading control. **B-C)** Total cellular RNA was isolated from differentiated 3T3-L1 cells treated as in

(A) for 24 h. The mRNA levels of **B)** ATG5 and **C)** ATG7 were quantified by real-time RT-PCR and normalized using internal control β-Actin. Values are mean ± SE of three independent experiments. Statistical significance relative to vehicle control, *$p < 0.05$.
